# Transcriptome analysis of the Bactrian camel (*Camelus bactrianus*) reveals candidate genes affecting milk production traits

**DOI:** 10.1186/s12864-023-09703-9

**Published:** 2023-11-02

**Authors:** Huaibing Yao, Zhihua Dou, Zhongkai Zhao, Xiaorui Liang, Haitao Yue, Wanpeng Ma, Zhanqiang Su, Yuzhuo Wang, Zelin Hao, Hui Yan, Zhuangyuan Wu, Liang Wang, Gangliang Chen, Jie Yang

**Affiliations:** 1 Key Laboratory of Biological Resources and Genetic Engineering, College of Life Science and Technology, Xinjiang University, 777 Huarui Street, Urumqi, 830017 Xinjiang PR China; 2 Xinjiang Camel Industry Engineering Technology Research Center, Urumqi, 830017 China; 3 College of Veterinary Medicine, Xinjiang Agricultural University, Urumqi, 830052 China; 4 Xinjiang Altai Regional Animal Husbandry Veterinary Station, Altay, 836500 Xinjiang China; 5 Bactrian Camel Academy of Xinjiang, Xinjiang Wangyuan Camel Milk Limited Company, Altay, 836500 Xinjiang China

**Keywords:** Bactrian camel, Milk production traits, Diagnosis of pregnancy, RNA sequencing, Milk-related genes

## Abstract

**Background:**

Milk production traits are complex traits with vital economic importance in the camel industry. However, the genetic mechanisms regulating milk production traits in camels remain poorly understood. Therefore, we aimed to identify candidate genes and metabolic pathways that affect milk production traits in Bactrian camels.

**Methods:**

We classified camels (fourth parity) as low- or high-yield, examined pregnant camels using B-mode ultrasonography, observed the microscopic changes in the mammary gland using hematoxylin and eosin (HE) staining, and used RNA sequencing to identify differentially expressed genes (DEGs) and pathways.

**Results:**

The average standard milk yield over the 300 days during parity was recorded as 470.18 ± 9.75 and 978.34 ± 3.80 kg in low- and high-performance camels, respectively. Nine female Junggar Bactrian camels were subjected to transcriptome sequencing, and 609 and 393 DEGs were identified in the low-yield vs. high-yield (WDL vs. WGH) and pregnancy versus colostrum period (RSQ vs. CRQ) comparison groups, respectively. The DEGs were compared with genes associated with milk production traits in the Animal Quantitative Trait Loci database and in Alashan Bactrian camels, and 65 and 46 overlapping candidate genes were obtained, respectively. Functional enrichment and protein–protein interaction network analyses of the DEGs and candidate genes were conducted. After comparing our results with those of other livestock studies, we identified 16 signaling pathways and 27 core candidate genes associated with maternal parturition, estrogen regulation, initiation of lactation, and milk production traits. The pathways suggest that emerged milk production involves the regulation of multiple complex metabolic and cellular developmental processes in camels. Finally, the RNA sequencing results were validated using quantitative real-time PCR; the 15 selected genes exhibited consistent expression changes.

**Conclusions:**

This study identified DEGs and metabolic pathways affecting maternal parturition and milk production traits. The results provides a theoretical foundation for further research on the molecular mechanism of genes related to milk production traits in camels. Furthermore, these findings will help improve breeding strategies to achieve the desired milk yield in camels.

**Graphical Abstract:**

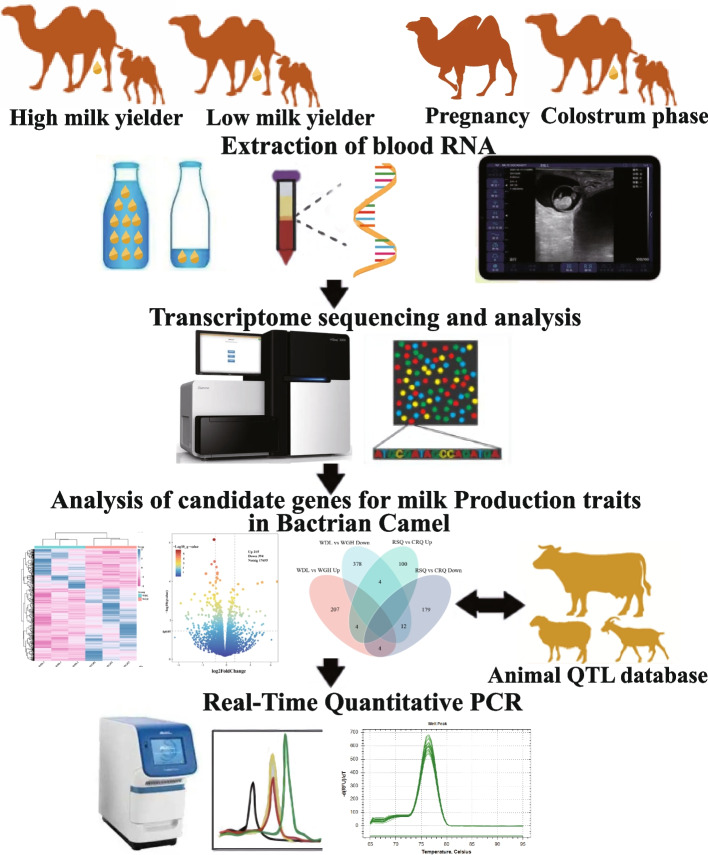

**Supplementary Information:**

The online version contains supplementary material available at 10.1186/s12864-023-09703-9.

## Introduction

 Camel is a unique domesticated species with an important role in arid and semi-arid animal husbandry and agriculture. Camels are used for service and the production of milk, meat, and wool. Through evolution, camels have developed unique abilities and attributes that enable their survival in extreme environments, including cold or hot arid regions with poor grazing [1, 2]. They produce heavy-chain antibodies, can withstand extreme hunger and thirst for long periods, and can consume toxic diets. According to the 2022 Food and Agriculture Organization of the United Nations database, approximately 38 million camels exist worldwide. Domestic dairy camels include the dromedary, distributed in the arid deserts of North and East Africa and Asia, and Bactrian camels, which inhabit the semi-arid desert regions of Asia [3]. In recent years, the demand for camel milk and milk products has increased both locally and internationally [4]. With an average annual milk production of approximately 3.1 million tons, camels rank fifth in milk production worldwide after cows, buffalo, goats, and sheep [5, 6]. Numerous genetic studies have investigated genes related to milk production traits in cows and sheep, but few studies have assessed milk production performance in camels.

Yield and composition are the most important economic traits of dairy camel milk. Milk is an emulsion of fat and water that contains dissolved carbohydrates, proteins, vitamins, and minerals. Nutrients in the blood must be converted into milk components by mammary epithelial cells [7]. Camel milk, which contains a high percentage of mono- and polyunsaturated fatty acids, has a healthier atherogenicity index than that from other animals [8] and unique therapeutic features [9]. Additionally, camel milk has a slightly higher concentration of immunity-enhancing, antibacterial, and insulin-like factors [10, 11]. The daily milk yield of Bactrian camels ranges from 1.5 - 5 kg (excluding the amount of milk consumed by young camels) over a lactation period of 10 to 12 months. This yield is considerably lower than that of dromedaries, which ranges from 3 - 10 kg [12], depending on breed, forage, lactation stage, and management conditions. Previous studies on milk traits in camels have mainly focused on physiological changes and biochemical indicators in milk and blood; few studies have investigated gene expression in relation to milk traits. To date, only a few studies have reported the association between *Casein* gene (*CSN2* and *CSN3*) polymorphisms and milk composition traits in camels [13–17].

Mammary gland tissue sampling is difficult, costly, and detrimental to animal welfare. Blood is easier to sample than other tissues as it requires limited animal handling. Moreover, blood reflects milk traits more directly and comprehensively than other tissue types [18]. A previous study of dromedary camel populations analyzed blood whole-genome sequencing and genotyping-by-sequencing data and identified genomic regions under positive selection that host genes potentially associated with milk content, including the phosphatidylinositol binding clathrin assembly protein (*PICALM*) gene [19]. Many studies have used blood transcriptome profiles to identify associations between gene expression and milk traits [18, 20–22]. The genes involved in milk synthesis could be differentially expressed in blood, and whole blood transcriptomics can be used as a non-invasive method to identify genetic variants that can reveal complex physiological interactions among various tissues during lactation [22]. A recent study performed a weighted correlation network analysis of the transcriptome profiles of lactating Alashan Bactrian camels under a supplemented grazing regime. Seven modules of approximately 1185 genes related to milk production were identified [23]. However, none of these genes have been verified.

Camels are typical seasonal breeders that inhabit low-productivity ecosystems. The gestation period of Bactrian camels is up to 400 days, and all camels give birth to a single calf. Camels usually reach sexual maturity at approximately 4 or 5 years of age [24]. Owing to the physiological characteristics and unique geographical distribution of Bactrian camels, the Bactrian camel breeding industry continues to employ the traditional pastoral system, with relatively primitive feeding methods and technologies and a lack of breeding information. To improve this situation, novel selection and breeding techniques are necessary. RNA sequencing (RNA-seq) has been widely used to identify notable functional genes associated with economically important production traits in animals. High-throughput sequencing can be used to identify novel genes that may serve as important markers for selecting camels with optimal milk production traits.

 This study aimed to identify genes related to lactation initiation and Bactrian camel milk production traits. Early pregnancy was detected using ultrasound, and blood was collected during gestation and the colostrum period for transcriptome sequencing. Low- and high-yield camels were subjected to whole-blood transcriptome sequencing, and gene expression profiles were compared between low- and high-yield camels and between pregnant camels and camels in the colostrum period.

## Materials and methods

### Camels and sampling sites

First, transcriptome sequencing was performed on a population comprising six unrelated domestic healthy Junggar Bactrian camels (Supplementary Fig. 1), including three low-yield (WDL, *n* = 3) and three high-yield camels (WGH, *n* = 3). The free-ranging camels were maintained under the same traditional grazing conditions in Fuhai County (88°24′11″ E, 47°9′8″ N), Altay Prefecture, Xinjiang Uygur Autonomous Region, Northwest China. These camels were 10 years old (fourth parity), with brown fluff and a weight range of 498–510 kg. They were selected based on milk production, as follows: high yielders, average milk yield approximately 3 kg/day/camel; low yielders, average milk yield approximately 1.5 kg/day/camel. Blood samples were collected via jugular vein puncture at peak lactation. In the second part of the study, three additional female camels were intensively reared for pregnancy (RSQ) and colostrum (CRQ) analysis at the Camel Dairy Farm in Jeminay County (86°12′9″ E, 47°40′34″ N). Blood samples were collected during gestation and the colostrum period (Supplementary Fig. 2). In addition, samples for microscopic observation of mammary tissue of pregnant and colostrum female camels were collected at local slaughterhouses (Supplementary Fig. 3). Genetically verified camels were obtained from Qibal Town in Habahe County (86°20′56″ E, 48°3′4″ N). All animal experiments were carried out according to the ethical guidelines of the Institution Review Board of Xinjiang University and were approved by the Ethics Committee of Xinjiang University (approval no.: XJU2019012). Samples were collected under license from the Guideline for Care and Use of Laboratory Animals of China. The milk yield and composition data are shown as the mean ± standard deviation (SD), and differences were determined via Student’s t-test using SPSS (version 26.0).

### Experimental design and sample collection

The low- and high-yield dairy camels included in this study were selected based on lactation data recorded during the 300-day milk production period in a single year (2020–2021). We evaluated the reproductive tracts of the camels, using transrectal ultrasonography with a 6.5 MHz rectal linear probe (GANDAOFU,GDF-C60) [25]. Transabdominal ultrasonographic detection and pregnancy evaluation were performed using a 5.0 MHz convex array probe. Pregnancy examination was conducted using a previously reported rectal ultrasound scan procedure designed for imaging the equine and camel gestational sac [26, 27]. Colostrum is produced by parturient female camels in the first 7 days after birth. Morphological observation of mammary tissue collected during pregnancy and the colostrum period was conducted using hematoxylin and eosin (HE) staining. All detailed manipulations were based on previous studies of cow and mouse mammary glands [28, 29]. Whole blood was collected into PAXgene Blood RNA Tubes (Qiagen) through venipuncture of the neck vein. The blood was immediately mixed by gently inverting the tube 10 times. These blood collection tubes contain special reagents to quickly protect the RNA in the cells. The tubes were left at room temperature on a bench overnight and subsequently stored on dry ice for shipping until use. All animal procedures were conducted in strict accordance with the Animal Ethics Guidelines and regulations of the People’s Republic of China.

### RNA isolation, library preparation, and sequencing

Total RNA was extracted using TRIzol reagent (Invitrogen, USA), according to the manufacturer's protocol. RNA integrity was assessed using the RNA Nano 6000 Assay Kit with the Bioanalyzer 2100 system (Agilent Technologies, USA) and 1.0% agarose gel electrophoresis. Novogene Co. Ltd. (Tianjin, China) performed cDNA library preparation and RNA-seq, generating 150 bp paired-end reads on the Illumina HiSeq 6000 platform [30]. Clean reads were obtained from raw data by removing low-quality reads and reads containing adapters or poly-N. The Q20, Q30, and GC contents of the clean data were then calculated. All downstream analyses were performed using clean, high-quality data. The index of the *Camelus bactrianus* reference genome was built using Hisat2 (version 2.0.5), and paired-end clean reads were aligned to the reference genome using Hisat. Feature Counts (version 1.5.0-p3) was used to count the reads mapped to each gene [30]. Gene expression values were calculated as fragments per kilobase of transcript per million mapped reads (FPKM).

### Differentially Expressed Gene (DEG) analysis

DEGs were identified between different comparison groups (WDL vs WGH, RSQ vs CRQ) using the DESeq2 package in R (version 4.2.1) based on negative binomial distribution. Considering previous studies [31, 32] and our specific experimental situation, adjusted *P*-values (*P*-adjust) < 0.05 and |log2FoldChange|> 0 were established as the thresholds for significance. Adjusted *P*-values were calculated using the Benjamini–Hochberg method.

### Comparative analysis of DEGs and the animal Quantitative Trait Loci (QTL) database

To limit the scope of the screening process and improve analysis accuracy, DEGs were compared with genes associated with the milk production traits (300-day milk yield, milk fat percentage, milk protein percentage, and milk protein content) of cattle, sheep, and goats in the Animal QTL database (https://www.animalgenome.org/cgi-bin/QTLdb). Additionally, a preprint study reported 1185 unvalidated candidate genes associated with the milk production traits of Alashan Bactrian camels [23], and we compared the DEGs identified in our study with these candidate genes.

### Functional enrichment analysis

To identify functions and pathways associated with the DEGs, Gene Ontology (GO) and Kyoto Encyclopedia of Genes and Genomes (KEGG) pathway enrichment analyses [33–35] were performed using the NovoMagic Cloud Platform (https://magic.novogene.com/customer/main#/loginNew). A cut-off of *P* < 0.05 was used to screen significant functions and pathways. We further screened core pathways and candidate genes by consulting the published literature.

### Protein–protein interaction network analysis

Gene regulatory networks elucidate how animal mammary glands are regulated during pregnancy and lactation [36]. Therefore, we mapped the identified DEGs into the Search Tool for the Retrieval of Interacting Genes (STRING) database (http://string-db.org/) [37]. Protein–protein interaction (PPI) networks were established using the Cytoscape software (version 4.8.0). Hub genes were identified using the Cytoscape plugins CytoNCA and CytoHubba [38].

### Quantitative real-time PCR analysis

The Prime Script RT Reagent Kit (Takara Biotechnology Co., Ltd.) was used to produce cDNA from the qualified RNA. The obtained cDNA was analyzed immediately or stored at -20 ℃. Camel beta-actin (*β-actin*) (NCBI Accession No. XM_010965866.2) was used as the internal reference gene [39]. Ten genes were selected from the DEGs between the low-yield and high-yield groups, and five genes were selected from the DEGs between the gestation and colostrum period groups. PCR was performed using the following cycling conditions: 95 °C for 5 min; 40 cycles of 95 °C for 10 s and 60 °C for 30 s; 95 °C for 15 s, 60 °C for 60 s, and 95 °C for 15 s. Specific primers were designed using the Primer-BLAST online website (Table 1, Supplementary Fig. 5). Expression levels were defined according to the threshold cycle, and the fold change in gene expression was calculated using the 2^−△△ct^ method [40]. Three biological replicates were established for each sample.

**Table 1:** Primer information for real-time quantitative PCR (*F* forward, *R* reverse)

Gene	Primer sequence (5’ → 3’)	Annealing temperature (°C)	Product length (bp)
*β-actin*	F: CAGATCATGTTCGAGACCTTC	55	275
	R: ATGTCACGCACGATTTCC		
*ABCA1*	F: CTCTCCTGTAGGAAGCAACCAG	56	129
	R: CCTTTGCCATCCATCCCACT		
*SLC8A1*	F: CGATGACTCTCCTTTTCTCC	54	168
	R: CTCCCAAGTTTCTTCTTGGA		
*LTF*	F: GAGAGATACTATGGCTACACTG	55	115
	R: GCTCAGTGTTCTTTCCATCAG		
*IGF2*	F: GGATCCCGATGGAGAAGT	55	160
	R: GGAAGTCTGAAGTAGAAGCC		
*NCKAP5*	F: CTGCTCACTCAAAAGGATCT	55	169
	R: GTGAGTGAAGTTTCTGGGTA		
*PGLYRP1*	F: TTAGGCACAGAAAGGTGAAG	56	180
	R: GCTCGTTTCACAGTTGAGTA		
*NR4A1*	F: TACGCTTATTTCTGGGTGAG	54	155
	R: AACCTCCTTCTTCTCTTCCT		
*SPP1*	F: ACCAAGGAAGAATCATCACC	55	132
	R: CCGGGTATTTGTTGTTAAGC		
*ARL4A*	F: AGTTGCTCTCACCAATAAGG	55	177
	R: GTTCACTCTGCTTAGCTCTT		
*KHDRBS3*	F: AGTGAAGAGGCTTACGATTC	54	123
	R: GTACAATCAGTATCTGCCGT		
*PARM1*	F: AACTCCGGGCTTTATGGAACA	58	101
	R: GGAAGAGAAACAGGTGAGAGG		
*HSPA12A*	F: GGAAACGGATACTGAGCTAC	55	81
	R: TTAGAAATCAGGGTTTGGCA		
*SRC*	F: CCAGATTGTCAACAACACGAG	57	70
	R: TCTTTGCAGCCATCTGAATGT		
*BAG2*	F: ACTAGTTCTTTCTGTTAGGCG	56	75
	R: ACACTGGAATATTTTCCTCGC		
*NLRP9*	F: TAAACTCCAGAAGTTCGTGT	55	192
	R: CCCAGCATCAGCTTTTCTAT		

## Results

### Identification of milk production traits

A summary of the statistics describing 300-day milk yield, fat percentage, and protein percentage is shown in Table 2 and Supplementary Table 1. A notable difference was observed between high-yield (978.34 ± 3.80 kg/300 days) and low-yield camels (470.18 ± 9.75 kg/300 days) (*P* < 0.001) maintained under the same feeding and milking conditions. The fat and protein percentages of high-yield camel milk were significantly lower than those of low-yield camel milk (*P* < 0.05). The milk production traits of lactating Bactrian camels with similar biological backgrounds differed significantly between groups, whereas consistency was observed within groups.

**Table 2:** Milk yield and components for different milk-producing groups

Group	Milk trait					
	300-day milk yield(kg)		Fats(g/100 g)		Protein(g/100 g)	
	Mean (± SD)	*P*-value	Mean (± SD)	*P*-value	Mean (± SD)	*P*-value
High milk yield (WGH1 ~ WGH3)	978.34 ± 3.80	*P* < 0.001	5.25 ± 0.04	*P* < 0.05	4.06 ± 0.01	*P* < 0.05
Low milk yield (WDL1 ~ WDL3)	470.18 ± 9.75		5.33 ± 0.01		4.11 ± 0.01	

**Fig. 1:**
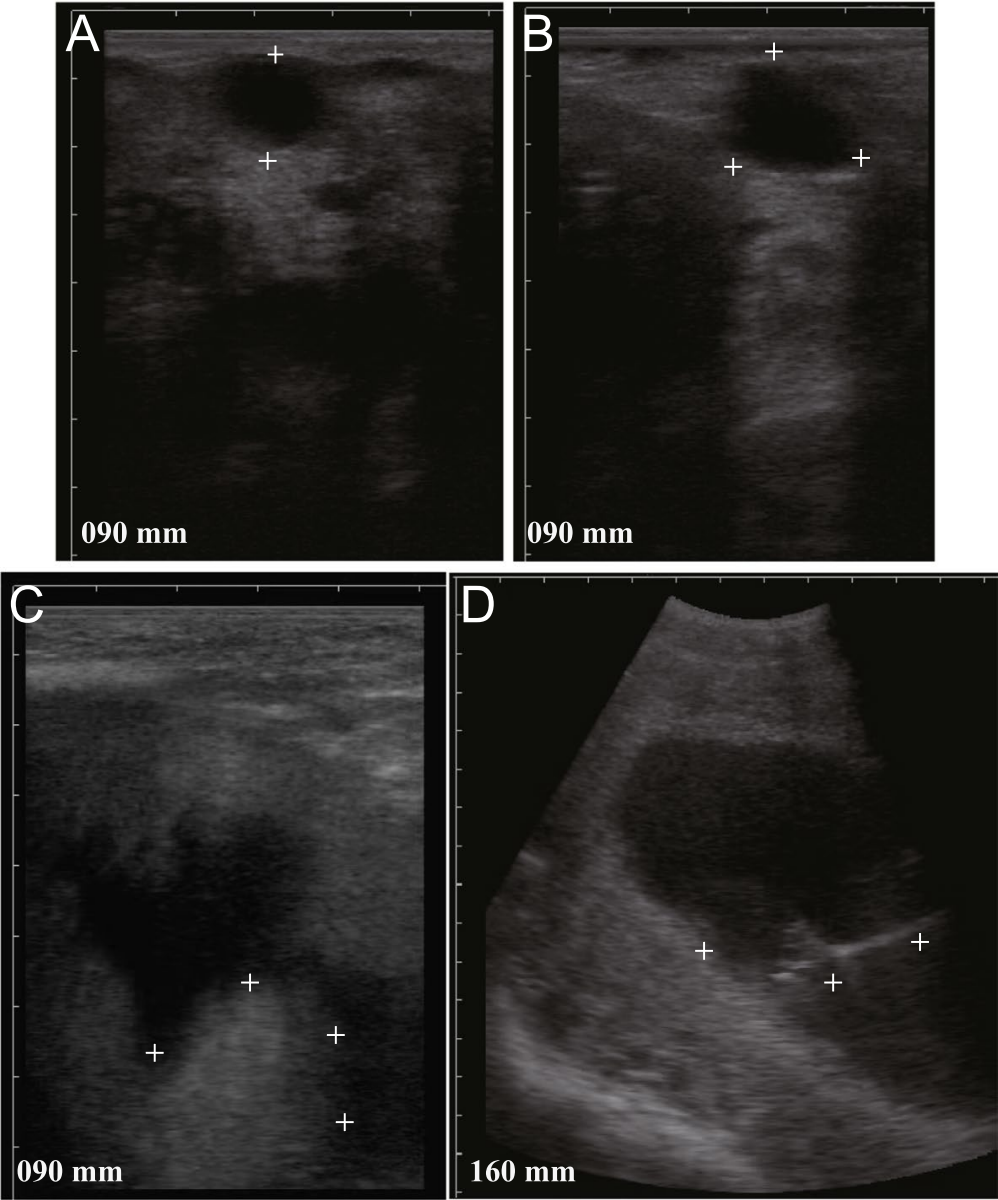
Ultrasound images of gestational sacs in Bactrian camel. **A** Transrectal ultrasonographic image of camel pregnancy 17 days after ovulation (6.5 MHz); **B** B-ultrasound imaging of camels at ~ 21 days of gestation (6.5 MHz); **C** B-ultrasound imaging of camels at ~ 90 days of gestation (6.5 MHz); **D** Transabdominal ultrasonographic images of camels at ~ 150 days of gestation (5.0 MHz)

**Fig. 2:**
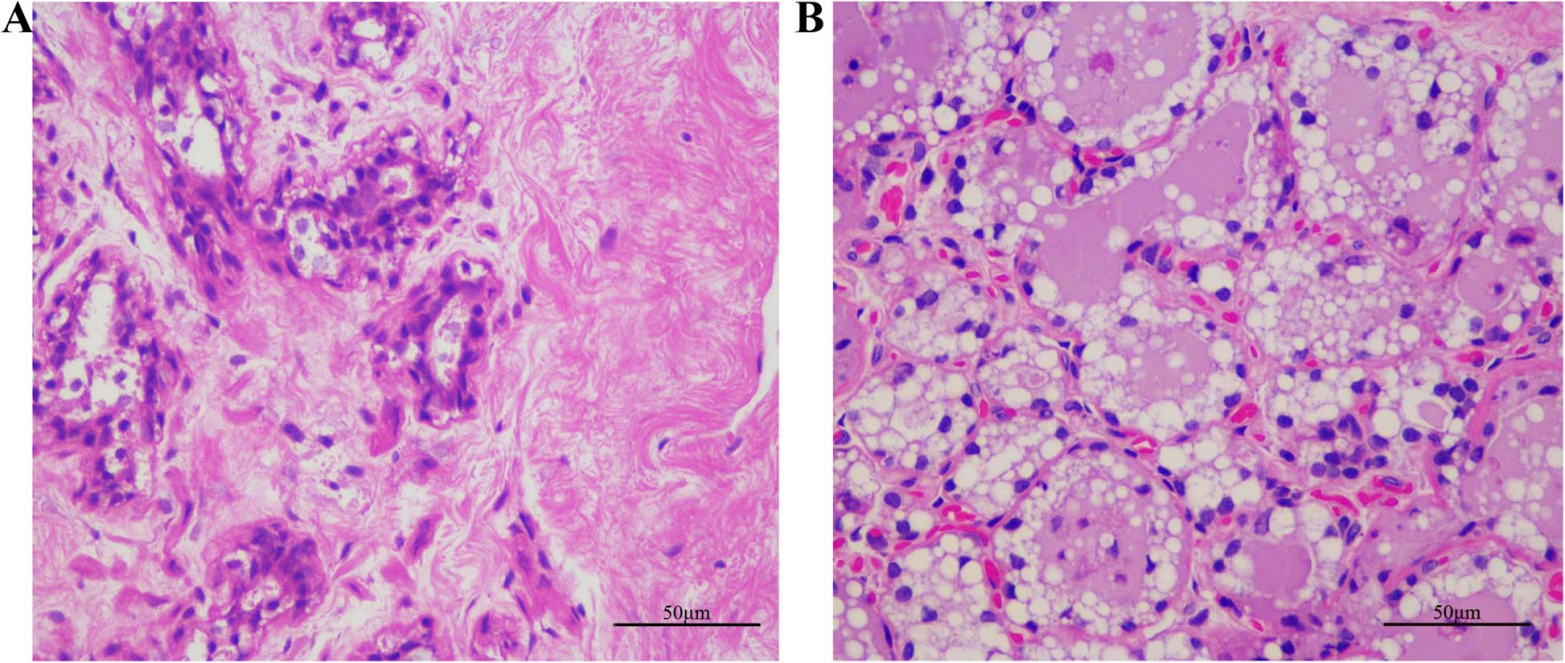
Microstructure of the mammary gland of the Bactrian camel. **A** Microstructure of pregnant mammary gland in camels (400 ×). **B** Microstructure of mammary gland in colostrum camels (400 ×)

The initial ultrasound of the embryonic vesicle in the uterine corpus revealed an all-black round or oval shape in the body of the uterus (Fig. 1A). As the intrauterine gestational sac developed, its shape changed from round or oval to an irregular triangle (Fig. 1B). This was caused by hyperplasia of the uterine wall at the back of the gestational body, which compressed the gestational sac into a triangular shape that was faintly visible and gradually became more prominent. This gestational image was consistent with the ultrasound images of horses [39] and donkeys [41] at 21 days of gestation, in which the embryo is initially visible at the base of the vesicle. At approximately 90 days of gestation, the gestational sac developed, enlarged, and sank into the abdominal cavity below the anterior border of the pelvic cavity (Fig. 1C). A visible difference was observed between the amniotic fluid surrounding the fetus and the larger volume of external urinary bladder fluid. Between 90 and 100 days, the conceptus could be easily identified using transabdominal ultrasonography (Fig. 1D). Ultrasonography provides an accurate visualization of early pregnancy in camels and can be used in commercial breeding. Regarding the colostrum period, there is a consensus that this stage occurs within 7 days of birth in domestic animals. Microscopic images of breast tissue at approximately 60 days of gestation revealed that the mammary duct system and vesicles grew and developed rapidly during pregnancy (Fig. 2A). These observations are generally comparable to those associated with cows [42]. During pregnancy, mammary epithelial cells proliferate and differentiate. The amount of fat and connective tissue increases, as does the number of capillaries. In this study, the mammary gland of the female camel became fully developed during the colostrum period, with several mammary vesicles and little intervesicular connective tissue (Fig. 2B).

### Overview of sequenced RNA

Supplementary Table 2 summarizes the quality of sequencing data collected from the two groups. The number of clean reads for each sample ranged from 40.50 - 51.64 million after the raw data were filtered. The percentage of mapped reads was above 88.23%, and the percentage of reads mapped to multiple positions on the reference genome was less than 2.5%. Q20 exceeded 97.76%, and Q30 exceeded 94.03%. The sequencing quality was high, and valid sequences were further analyzed . All sequencing results were uploaded and deposited in the NCBI Sequence Read Archive database under accession numbers PRJNA892805 and PRJNA892714.

### Analysis of DEGs

**Fig. 3:**
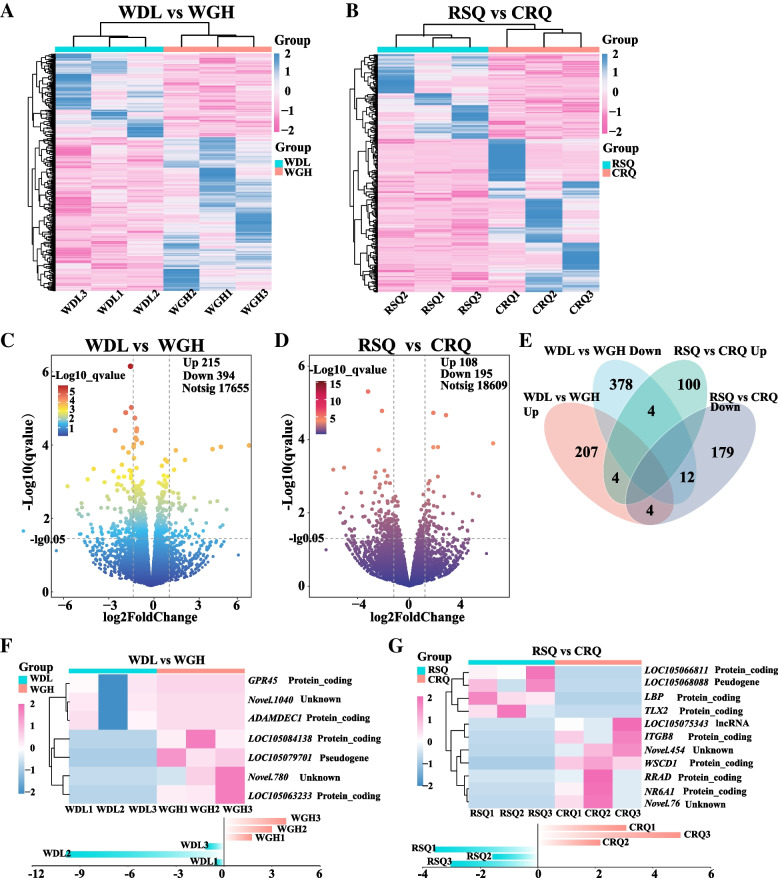
Differentially expressed genes (DEGs) between low-yield and high-yield, gestating and primiparous camels. **A** Heatmap of DEGs in low-yield vs. high-yield lactating camels (WDL vs. WGH); **B** Heatmap of DEGs in the pregnant and colostrum female camels (RSQ vs. CRQ); **C** Volcano plot of DEGs (WDL vs. WGH); **D** Volcano plot of DEGs (RSQ vs. CRQ); **E** Venn diagram of the comparison of DEGs in the four groups of samples; **F** Analysis of specific DEGs (WDL vs. WGH); **G** Analysis of specific DEGs (RSQ vs. CRQ)

Principal component analysis (PCA) confirmed that intra-group camel samples exhibited a certain correlation (Supplementary Fig. 4). The FPKM values of the RNA library samples were clustered using the mainstream hierarchical clustering method, and row data were converted into Z-scores. As shown in the clustering heatmaps (Fig. 3A and B), prominent differences were observed in the expression patterns of DEGs between the two groups. A total of 609 and 393 DEGs were identified in the low-yield vs. high-yield (WDL vs. WGH) and pregnancy vs. colostrum period (RSQ vs. CRQ) groups, respectively (Fig. 3C and D, Supplementary Tables 3 and 4). Four common DEGs were significantly upregulated, and 12 common DEGs were significantly downregulated (Fig. 3E). Eight genes exhibited inconsistent differential expression trends. Among the 24 genes, lactotransferrin (*LTF*), nuclear receptor subfamily 4 group A member 1 (*NR4A1*), and polymeric immunoglobulin receptor (*PIGR*) have been associated with milk production traits in domestic animals. Eight DEGs were identified between low-yield and high-yield camels, as shown in Fig. 3F. Three of these DEGs were expressed in the low-yield group but not in the high-yield group; the other five DEGs exhibited the opposite trend (Supplementary Table 5). In the pregnancy and colostrum groups, 11 stage-specific genes were identified (Fig. 3G). Among them, four were expressed in the gestation group alone, and seven were expressed in the colostrum group alone. Most of these genes were protein-coding genes that may regulate certain biological functions.

### Comparative analysis of DEGs and Animal QTL database genes

**Fig. 4:**
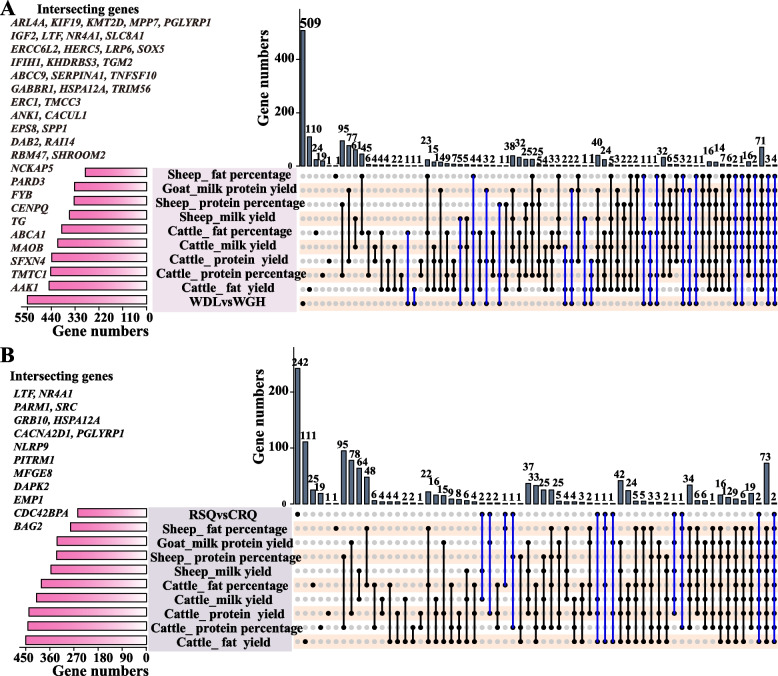
Common genes between DEGs and animal QTL database milk traits genes. **A** Upset plot of milk trait and camel DEGs (WDL vs. WGH) common genes in the animal QTL database; **B** Upset plot of milk trait and camel DEGs (RSQ vs. CRQ) common genes in the animal QTL database

The DEGs of low-yield and high-yield Bactrian camels were compared with genes in the Animal QTL database, and 42 overlapping genes associated with milk production traits in domestic animals were identified (Fig. 4A). Of these genes, expression levels of 12 genes were upregulated and those of 30 genes were downregulated (Supplementary Table 6). These included insulin-like growth factor 2 (*IGF2*), *LTF*, *NR4A1,* and solute carrier family 8 member A1 (*SLC8A1*), which are closely associated with milk production traits in cattle, goats, and sheep. Furthermore, the DEGs of pregnant and colostrum Bactrian camels were compared with genes in the Animal QTL database, and 15 overlapping genes associated with milk production traits in domestic animals were identified (Fig. 4B). Among them, expression levels of six genes were upregulated, and those of nine genes were downregulated (Supplementary Table 6). These included *LTF* and *NR4A1*, which are closely associated with milk production traits in cattle, goats, and sheep. The DEGs were also compared with candidate genes associated with Alashan Bactrian camel milk traits [23], and 17 (WDL vs. WGH) and 21 (RSQ vs. CRQ) overlapping genes were identified (Table 3, Supplementary Table 7). All overlapping genes were considered candidate genes and are available for downstream enrichment analysis. These findings confirm previous results and highlight associations between genes and milk production traits in different domestic animal species.

**Table 3:** Comparative analysis of differentially expressed genes and related genes to milk production traits in Alashan Bactrian Camel

Group	Number of common genes	List of common genes	Reference
WDL vs WGH	17	↑*TMEM119*, ↓*LOC105082373*, ↓*LOC105079701*, ↑*EGFL7*, ↑*ADAMDEC1*, ↑*CERCAM*, ↑*TDO2*, ↑*LOC105080024*, ↓*LOC105066866*, ↑*SBSN*, ↑*EHD2*, ↑*LOC105083746*, ↑*LOC105065387*, ↑*GCSAM*, ↓*OAS1,* ↓*GLYATL3*, ↓*LOC105083358*	[23]
RSQ vs CRQ	21	↓*LBP*, ↓*GRIP1*, ↓*CACNA2D1*, ↑*LOC105076976*, ↓*GGT1*, ↓*KLRD1*, ↓*EHD2*, ↓*WSCD1*, ↓*LOC105074728*, ↓*LOC105062155*, ↓*GGT5*, ↑*LOC105080024*, ↓*CCDC83*, ↓*CPT1B*, ↓*LOC105080037*, ↓*LOC105077107*, ↓*DSCAML1*, ↑*S100B*, ↑*CDHR1*, ↓*ARHGAP29*, ↑*TLX2*	

The upward arrow indicates that the genes are up-regulated; and the downward arrow, down-regulated

### Functional enrichment analysis

**Fig. 5:**
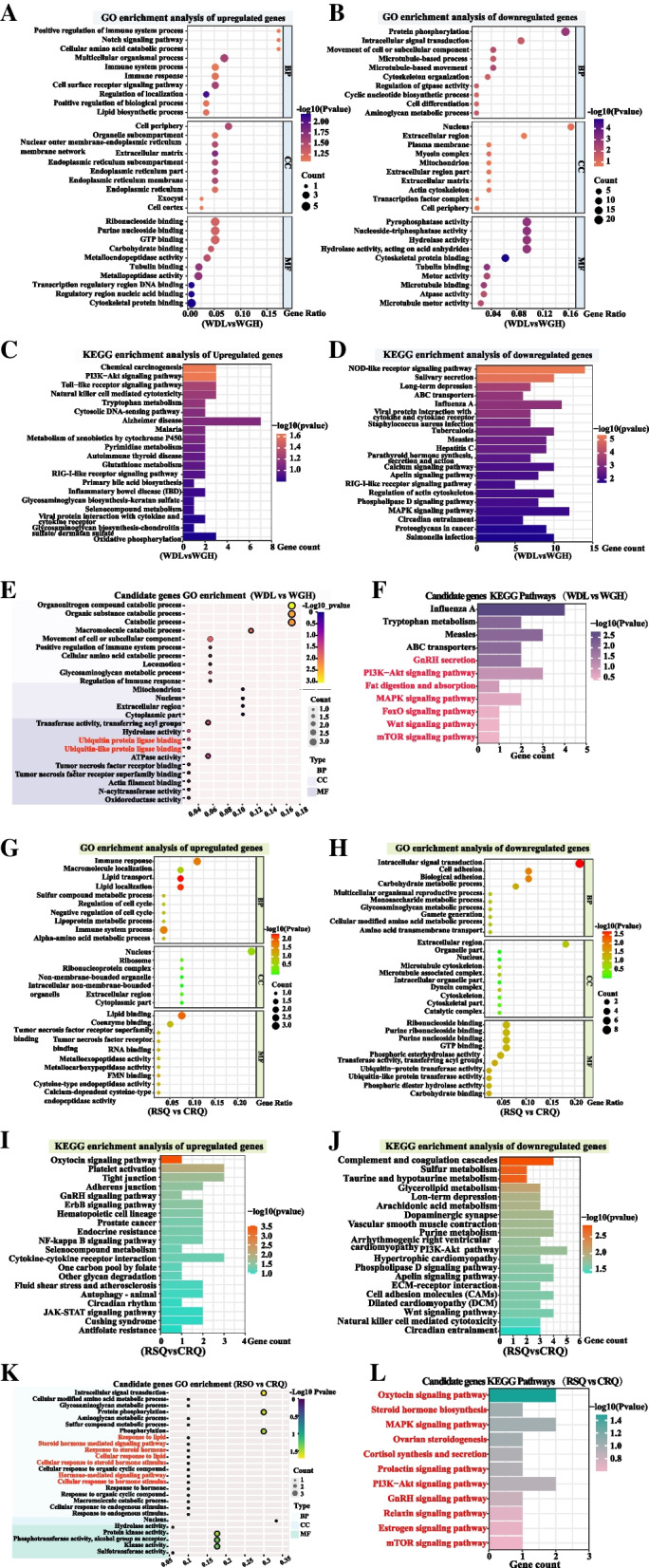
Functional enrichment analysis of differentially expressed genes. **A**-**F** WDL vs. WGH: **A** GO enrichment analysis of upregulated genes in camels between the low-yield and high-yield groups; **B** GO enrichment analysis of downregulated genes in camels between the low-yield and high-yield groups; **C** KEGG pathway analysis of upregulated genes in camels between the low-yield and high-yield groups; **D** KEGG pathway analysis of downregulated genes in camels between the low-yield and high-yield groups; **E** GO enrichment analysis of 65 candidate genes; **F** Pathway enrichment analysis of 65 candidate genes. **G**-**L** RSQ vs. CRQ: **G** GO enrichment analysis of upregulated genes in camels between the pregnancy and colostrum groups; **H** GO enrichment analysis of downregulated genes in camels between the pregnancy and colostrum groups; **I** KEGG pathway analysis of upregulated genes in camels between the pregnancy and colostrum groups; **J** KEGG pathway analysis of downregulated genes in camels between the pregnancy and colostrum groups; **K** GO enrichment analysis of 46 candidate genes; **L** Pathway enrichment analysis of 46 candidate genes

Functional enrichment analysis was conducted for upregulated or downregulated genes in each experiment. Functional enrichment analysis of DEGs in high- and low-yielding camels is shown in Supplementary Table 8. The top 10 GO term entries in each GO category were selected for display. The upregulated genes were primarily enriched in the GO entries related to lipid biosynthesis, immune-related responses, and cell-related biological processes (Fig. 5A). The downregulated genes were mainly enriched in intracellular signal transduction, the aminoglycan metabolic process, and transcription regulatory region DNA binding (Fig. 5B). In the KEGG pathway analysis, we showed the top 20 enriched pathways. KEGG pathway enrichment analysis revealed that the upregulated genes were significantly enriched in phosphoinositide-3 kinase-cellular homolog of the v-akt oncogene, an S/T protein kinase (PI3K–Akt), and chemical carcinogenesis (*P* < 0.05; Fig. 5C). The PI3K–Akt signaling pathway is involved in livestock milk yields [43, 44]. The downregulated DEGs were significantly enriched in 32 KEGG pathways (*P* < 0.05), including the mitogen-activated protein kinase (MAPK) signaling pathway, which has been previously reported to be associated with livestock milk yields (Fig. 5D). Subsequently, the DEGs were screened using multiple comparative analysis methods, and 65 candidate genes associated with milk production traits were identified (Supplementary Table 9). These genes were subjected to enrichment analysis, and 127 GO terms were yielded (Supplementary Table 10). The top 10 functional items per category were mainly associated with ubiquitination pathway, organic matter catabolic process, and immune system process (Fig. 5E). Of these, only four cellular component terms were enriched. The ubiquitination pathway plays a critical regulatory role in synthesizing milk fat [45]. The analysis also revealed five significantly enriched signaling pathways (*P* < 0.05), including gonadotropin-releasing hormone (GnRH) secretion, a vital metabolic pathway associated with animal milk production traits. The PI3K–Akt signaling pathway, fat digestion and absorption pathway, MAPK signaling pathway, forkhead box protein o (FoxO) signaling pathway, contraction of “Wingless” and “Int” (Wnt) signaling pathway, and mammalian target of rapamycin (mTOR) signaling pathway were also enriched, although not significantly; these pathways have been associated with milk production traits. Genes enriched in these pathways were also considered candidate genes (Fig. 5F) [46–50].

The DEGs between the pregnancy and colostrum groups were subjected to enrichment analysis (Table Supplementary Table 11). The upregulated genes were mostly enriched in the GO entries related to lipid transport, localization and binding, immune response, and regulation of the cell cycle (Fig. 5G). GO analysis of downregulated genes indicated predominant enrichment in intracellular signal transduction, the multicellular organismal reproductive process, and ubiquitin-protein or ubiquitin-like protein transferase activity (Fig. 5H). Subsequently, the upregulated genes were predominantly enriched in the oxytocin signaling pathway, GnRH signaling pathway, JAK–STAT signaling pathway and tight junction (Fig. 5I). The downregulated genes were enriched in the PI3K–Akt signaling pathway, Wnt signaling pathway, phospholipase D signaling pathway, and Apelin signaling pathway, among others (Fig. 5J). The functional enrichment results demonstrated that the DEGs have roles mainly in the Wnt signaling pathway, GnRH signaling pathway, tight junction, and oxytocin signaling pathway, which have been shown to be closely associated with parturition and lactation initiation in female animals [51–55]. The 46 candidate genes identified in pregnant and colostrum period camels were enriched in 61 GO categories and 64 signaling pathways (Supplementary Tables 12 and 13). Figure 5K shows the top 20 biological process terms, the top five molecular function terms, and the single cellular component term. Consistent with the GO enrichment analysis results, the predominant biological process terms included steroid hormones and lipid metabolism. Based on previous studies, 11 signaling pathways were screened from those identified via KEGG enrichment analysis (Fig. 5L). All terms and pathways were closely associated with maternal parturition, estrogen regulation, and initiation of lactation [56–60].

**Fig. 6:**
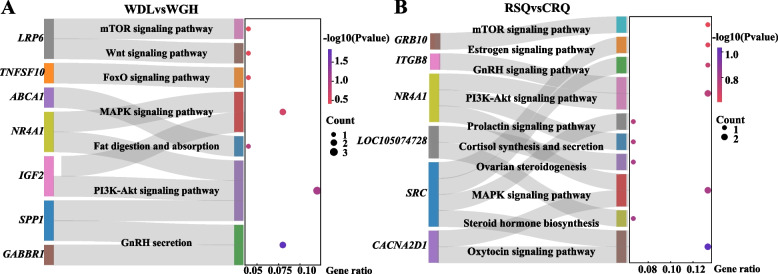
Sankey plot showcasing hub gene enrichment pathways in candidate genes. The dot plot showed the gene ratio between pathways and the number of genes in each enriched pathway. **A** hub gene enrichment pathways in candidate genes of low-yield vs. high-yield groups (WDL vs. WGH); **B** hub gene enrichment pathways in candidate genes of pregnancy vs. colostrum camel groups (RSQ vs. CRQ)

The KEGG enrichment analysis results showed that the candidate genes identified in the low-yield vs. high-yield and pregnancy vs. colostrum comparison groups were mainly involved in 7 and 11 KEGG pathways, respectively. Gamma-aminobutyric acid (GABA) B receptor 1 (*GABBR1*) and secreted phosphoprotein 1 (*SPP1*) were related to GnRH secretion; *SPP1*, *NR4A1,* and *IGF2* were associated with the PI3K-Akt signaling pathway; ATP binding cassette transporter 1 (*ABCA1*) was related to the fat digestion and absorption signaling pathway; *NR4A1* and *IGF2* were associated with the MAPK signaling pathway; tumor necrosis factor superfamily member 10 (*TNFSF10*) was related to the FoxO signaling pathway; and low density lipoprotein receptor-related protein 6 (*LRP6*) was associated with the Wnt and mTOR signaling pathways (WDL vs WGH) (Fig. 6A). Sarcoma (*SRC*) was related to oxytocin, prolactin, GnRH, and estrogen signaling pathway; *NR4A1* was correlated with the MAPK pathway, cortisol synthesis and secretion, and P13K-Akt signaling pathway; calcium channel, voltage-dependent, alpha 2/delta subunit 1 (*CACNA2D1)* was related to oxytocin and the MAPK signaling pathway; *L0C105074728* was related to steroid hormone biosynthesis and ovarian steroidogenesis; integrin beta-8 (*ITGB8*) was associated with the P13K-Akt signaling pathway; and growth factor receptor-bound protein 10 (*GRB10*) was associated with the mTOR signaling pathway (RSQ vs CRQ) (Fig. 6B).

### PPI network of DEGs

**Fig. 7:**
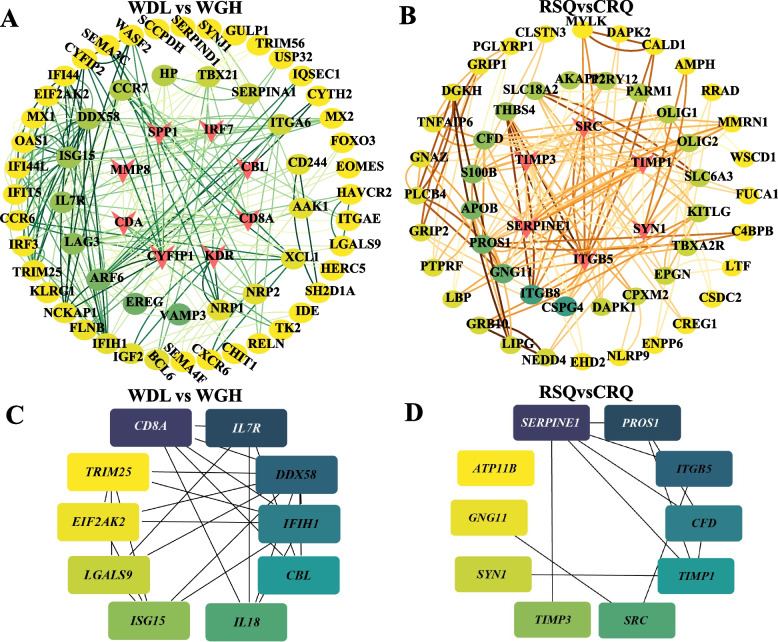
Protein–protein interaction (PPI) network analysis of differentially expressed genes. **A** Enriched network in the DEGs analysis using modules of CytoNCA in camels of low-yield vs. high-yield groups (WDL vs. WGH); **B** Enriched network in the DEGs analysis using modules of CytoNCA of pregnancy vs. colostrum camel groups (RSQ vs. CRQ); **C** Enriched network in the DEGs analysis using modules of CytoHubba in camels of low-yield vs. high-yield groups (WDL vs. WGH); **D** Enriched network in the DEGs analysis using modules of CytoHubba of pregnancy vs. colostrum camel groups (RSQ vs. CRQ)

By integrating the protein information in the STRING database with the DEGs, a PPI network was established. Disconnected nodes were deleted, and the network with the most nodes was retained. Based on the PPI network, kinase domain-containing receptor (*KDR*), cytoplasmic FMR1 interacting protein 1 (*CYFIP1*), cytidinedeaminase (*CDA*), matrix metallopeptidase 8 (*MMP8*), *SPP1*, interferon regulation factor 7 (*IRF7*), calcineurin-b like protein (*CBL*), and cluster of differentiation 8 alpha (*CD8A*) (Top8, Betweenness (BC) values > 1000) (Fig. 7A) were included in the high- and low-yield groups; plasminogen activator inhibitor type 1 (*SERPINE1*), tissue inhibitor of metalloproteinase 3 (*TIMP3*), *SRC*, tissue inhibitor of metalloproteinase 1 (*TIMP1*), synapsin I (*SYN1*), and integrin beta-5 (*ITGB5*) (Top6, BC values > 500) (Fig. 7B) were included in the pregnancy and colostrum groups. In addition, gene data were imported into CytoHubba, and key genes were identified using the degree calculation method. The top 10 genes were considered hub genes, and a degree of interaction was observed between some hub genes. Comparative analysis of the PPI networks for the low- and high-yield groups was conducted. *CD8A* and *CBL* were the common hub genes, and interleukin-7 receptor subunit alpha (*IL7R*), interferon stimulated gene 15 (*ISG15*), interferon induced with helicase C domain 1 (*IFIH1*)*,* tripartite motif-containing protein 25 (*TRIM25*)*,* and eukaryotic translation initiation factor 2-alpha kinase 2 (*EIF2AK2*) played substantial roles in the network (Fig. 7A and C). Comparative analysis of the PPI networks for the pregnancy and colostrum groups was performed. *SERPINE1*, *TIMP1*, *SRC*, *TIMP3,* and *SYN1* were the common hub genes, and protein s (alpha) (*PROS1*), complement factor d (*CFD*), and guanine nucleotide binding protein (G protein) gamma 11 (*GNG11*) played key roles in the network (Fig. 7B and D).

### Validation of gene expression

**Fig. 8:**
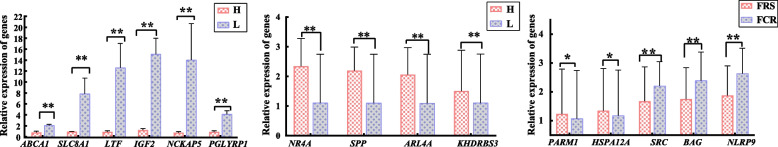
Expression level of DEG validation by qRT-PCR. H = high-yield camels; L = low-yield camels; FRS = pregnant camels; FCR = camels in colostrum stage; all data are presented as the Mean ± SD (*n* = 3); *P < 0.05; **P < 0.01

We selected 10 DEGs, including *ABCA1*, *SLC8A1*, *LTF*, *IGF2*, nck-associated protein 5-like (*NCKAP5*), peptidoglycan recognition protein 1 (*PGLYRP1*), *NR4A1*, *SPP1*, adp-ribosylation factor-like 4a (*ARL4A*), and KH domain containing RNA binding signal transduction associated 3 (*KHDRBS3*) from the RNA sequence data of low- and high-yield camels, and selected five DEGs, including prostate androgen-regulated mucin-like protein 1 (*PARM1*), heat shock 70kda protein 12a (*HSPA12A*), *SRC*, BCL2-associated athanogene 2 (*BAG2*)*,* and nucleotide-binding oligomerization domain leucine rich repeat and pyrin domain containing proteins (*NLRP*) from RNA sequence data of pregnant and colostrum female camels to verify their expression patterns using qRT-PCR in another regional population of Bactrian camels in Habahe County (Supplementary Table 14). The qRT-PCR results confirmed that the expression pattern of these genes was consistent with the RNA-seq (Fig. 8). These results confirm that the gene expression results of the blood transcriptome sequencing analysis are highly credible.

## Discussion

Female camels have only one parity per 2 years and give birth to only one fetus per pregnancy. Reproductive performance and milk production performance tend to increase simultaneously after maturity and decrease in older individuals [61]. Consequently, camel milk is extremely valuable because of its limited production, unique nutritional composition, and medicinal properties. The accurate diagnosis of pregnancy early after mating enables the efficient management of camel herds. Transrectal ultrasonography is the most accurate method for detecting early pregnancy. However, the pregnancy diagnosis reports in camels remain limited [27, 62]. In our study, ultrasonography of the embryonic vesicle served as a non-invasive method for the accurate visualization of dynamic changes during early pregnancy and the collection of basic diagnostic data. Lactation involves the dynamic process of mammary gland development and the synthesis and secretion of milk [63]. Development of lactation in the mammary glands follows a similar pattern to that of mammary gland development, with large amounts of follicular lobular tissue forming rapidly in the late stages of pregnancy [64]. After development during pregnancy, the morphology and structure of camel mammary tissue change significantly (Fig. 2). The mammary gland in the colostrum period matures with the function of milk production; however, the secretory tissue continues to proliferates rapidly in the late gestation and early lactation stages; the modifications that the epithelial cells undergo are required for milk production.

Milk production traits, including milk yield, fat yield, protein yield, fat percentage, and protein percentage, are quantitative traits influenced by combinations of genes, polygenes, and environmental factors. Milk protein variability can be used to study the associations concerning milk performance traits in camels [65]. Several studies have also reported that camels have high genetic variability due to the lack of selective breeding [66]. With respect to the milk production results, we found that even though there were no differences during the feeding conditions of camels in lactation, there were notable differences in the milk yield and composition. Understanding the genetic factors that influence animal reproductive and milk production traits is vital for accurate selection and the genetic improvement of quantitative traits [67]. In preliminary research, we wanted to explore detailed expression of genes in very different physiological stages and identify genetic factors associated with milk characteristics to facilitate the molecular genetic breeding of camels. Through transcriptome sequencing of camel whole blood, 609 and 393 DEGs were identified in low- vs. high-yield (WDL vs. WGH) and pregnancy vs. colostrum period (RSQ vs. CRQ) groups, respectively. This finding indicates that there are notable changes in gene expression at the transcriptional level during initiation and maintenance of the lactation process. Among these genes, some with high expression are related to milk yields, whereas others were found to be related to the milk composition. *LTF* is associated with milk yield in cattle and goats [68, 69]. *NR4A1* is significantly differentially expressed between the mammary glands of lactating cows with extremely high and low percentages of milk protein and fat [70]. *PIGR* (milk whey protein) is considered a biomarker for early pregnancy in dairy cows [71]. Additionally, we identified specifically expressed genes in the transcriptional profiles, some of which have been reported to be associated with growth traits, milk traits, reproductive performance, and mammary gland diseases in livestock [20, 21]. The expression of genes encoding production traits (*WSCD1*, *NR6A1*, *ITGB8*) was significantly absent during the pregnancy stage, as lactation does not occur during this period [72, 73]. Similarly, the *MTNR1A* gene is specifically expressed in the blood of high-yielding camels, and it encodes the receptor responsible for transducing melatonin signaling [74].

The Animal QTL database contains numerous significant chromosomal regions, and molecular markers associated with milk yield and composition have been identified. Among the screened DEGs and genes associated with livestock milk traits in the database, 42 and 15 overlapping genes were identified as candidate genes associated with milk production traits and initiation of lactation, respectively. Moreover, there were more downregulated genes than upregulated genes. Regarding these overlapping genes, many milk traits (fat, protein, and milk yield traits) are controlled by multiple genes, and some genes have pleiotropic effects on yield traits or other traits. Additionally, among the DEGs analyzed by sequencing, 17 (WDL vs. WGH) and 21 (RSQ vs. CRQ) DEGs were consistent with those identified by Guo et al. [23], who reported the transcriptome profiles of lactating Alashan Bactrian camels maintained under different feeding conditions. The results indicate that there are some common functional genes among different breeds of Bactrian camels. More similar studies are warranted to explore the functions of these genes.

To elucidate the underlying mechanisms of milk production, functional enrichment analysis of the DEGs was performed. GO analysis of the DEGs indicated that certain terms that play a key role in milk production were enriched, such as the multicellular organismal process, transcriptase activity factor, positive regulation of biological process, transcription factor complex, and actin cytoskeleton reorganization. The KEGG pathway enrichment analysis of the DEGs between low- and high-yield camels showed that some upregulated genes were involved in mammary gland physiology and belonged to the PI3K–Akt signaling pathway. The PI3K–Akt signaling pathway is a canonical pathway involved in mammary development and lipid metabolism [75]. Liu et al. [76] found that the lncRNA mammary proliferation and fatty acid synthesis-associated transcript promotes proliferation and fatty acid synthesis in bovine mammary epithelial cells by sponging miR-103, which regulates the PI3K–Akt pathway. Among the downregulated gene enrichment pathways, MAPK signaling pathways are related to higher milk yields in cattle [77]. The p38–MAPK pathway is a crucial signaling pathway that plays a critical role in mammary epithelial cell development and enhances milk production by modulating alveolar cell proliferation and branching [78]. Overall, these signaling pathways have vital roles in the proliferation and development of mammary epithelial cells and promote milk secretion and production through glucose uptake from the blood [79]. Further enrichment analysis of the candidate genes revealed that they were categorized into 127 GO categories and 82 signaling pathways, from which we selected two GO entries and seven signaling pathways. Previous studies have demonstrated an association between animal milk fat synthesis and the ubiquitination pathway, insulin (PI3K-Akt) pathway, Wnt signaling pathway, and mTOR signaling pathway [45–47, 49, 50]. Animal milk yield is closely linked to GnRH secretion, the MAPK signaling pathway, and the FoxO signaling pathway [43, 48, 80]. These results suggested that post-transcriptional and post-translational modifications play a determinative regulatory role in lactogenesis through energy homeostasis and by counteracting stress, favoring high milk production. The published literature provides robust support for our findings [22].

To gain insight into the milk production-related biological processes that occur during pregnancy and the colostrum period, we performed enrichment analyses. The GO analyses suggest that many of the DEGs are involved in terms related to cell development processes, the multicellular organismal reproductive process, and the metabolism of various substances and hormones. The partial downregulated genes are associated with ubiquitin-like protein transferase activity. It has been documented that ubiquitin-like proteins play a crucial role in the maintenance of the tissue cell cycle and lactation in animals [81]. KEGG pathway analyses of candidate genes yielded 11 signaling pathways that were closely related to maternal parturition, estrogen regulation, and initiation of lactation. Numerous published studies have reported that prepartum dietary energy intake is correlated with glycerolipid metabolism and that maternal parturition is associated with the oxytocin signaling pathway, steroid hormone biosynthesis, ovarian steroidogenesis, cortisol synthesis and secretion, the GnRH signaling pathway, the relaxin signaling pathway, and the estrogen signaling pathway [36, 53, 82, 83]. Milk production traits are linked to the MAPK, PI3K-Akt, GnRH, and mTOR signaling pathways [56–60]. A progressive increase in progesterone and serum estrogen levels is observed with advancing pregnancy. When estrogen stimulation leads to an increase in the number and size of lactotroph cells within the gland to a certain extent, it results in lactotrophic cell synthesis and prolactin secretion [84]. The mammary glands are sufficiently developed to produce these components owing to lactogen stimulation and regulation. Subsequently, myoepithelial cells respond to oxytocin and the contraction of milk-producing alveolar cells, and the female animal experiences a milk-ejection reflex [85]. Enriched pathways showed the remarkably intricate tuning of metabolic and cell development processes converging in milk production in camels. In summary, pregnant camels must have certain levels of progesterone, estrogen, and other steroid hormones to produce milk.

Pregnancy and lactation progress through tightly coordinated gene regulatory networks. These genes were also highlighted in the PPI network. We explored the potential biological function of common hub genes in the four PPI networks by searching published studies. Among these genes, *CD8A*, *CBL*, *SERPINE1*, *TIMP1*, *SRC*, *TIMP3*, and *SYN1* were the common hub genes in the four PPI networks, and *IL7R*, *ISG15*, *IFIH1*, *TRIM25*, *EIF2AK*, *PROS1*, *CFD*, and *GNG11* had significant network roles. We believe that the overlapping genes identified via different analysis methods are hub genes associated with maternal parturition, estrogen regulation, initiation of lactation, and milk production traits. *GABBR1*, *SPP1*, *ABCA1*, *NR4A1*, *IGF2*, *TNFSF10*, *LRP6*, *CACNA2D1*, *L0C105074728*, *ITGB8*, *SRC,* and *GRB10* were identified as core DEGs. The complex relationships among these core genes suggest that milk production traits are quantitative traits regulated by multiple hormones and pathways. We identified 16 signaling pathways and 27 core candidate genes associated with maternal parturition, estrogen regulation, lactation initiation, and milk production traits in camels. Some are genes with unknown function. In the present study, 15 DEGs were selected for qPCR to verify the RNA-seq data. The expression patterns obtained by qRT-PCR and RNA-seq were similar, indicating the reliability of the transcriptome results. The identification of these genes will contribute to the knowledge regarding the molecular mechanisms associated with milk production traits for lactating camels.

The enrichment of these functions suggests that lactation is the result of the mutual control of signals induced by metabolites/molecules derived from multiple organs and tissues, in synergy with the regulation of immune functions in camels [22]. Candidate genes associated with milk production traits in ruminants have been thoroughly investigated, but little information about camels is available. Therefore, this study may serve as a foundation for further genetic studies of camel milk production traits. Nevertheless, one of the limitations of this study was the sample size, which was affected by objective factors, such as small-scale free-ranging camel farming, a low breeding technology level, and challenges associated with the measurement of milk production performance and sampling, which were difficult and time-consuming. Some of the 27 core candidate genes identified here have not previously been associated with milk production traits, and further in-depth research is necessary. The further genetic identification of these candidate genes could lead to their utilization as important markers for selecting superior camels. Owing to the lack of data and complexity of the interactions among genes, large-scale samples will be required in the future to fully elucidate the associations between these genes and milk production traits in Bactrian camels. In conclusion, this study is of great value for identifying critical genes that control the initiation of lactation and milk production traits and can inform subsequent studies on the molecular mechanisms underlying lactation in camels.

## Conclusions

Our study is the first to examine pregnant Bactrian camels using B-mode ultrasonography; in addition, the blood transcriptome profiles of camels in low-yield, high-yield, pregnant, and colostrum period groups were reported. Gene expression patterns related to cell growth, hormone regulation, lipid metabolism, immunity, material transport, biosynthesis, and metabolism were considerably altered during mammary gland development and lactation. We identified 16 core signaling pathways and 27 core candidate genes associated with maternal parturition, estrogen regulation, initiation of lactation, and milk production traits. These promising genetic markers could be informative for identifying quantitative trait loci associated with milk production traits and developing markers for controlled camel breeding programs. This study identified candidate genes and signaling pathways that affect maternal parturition and milk production traits, establishing a theoretical foundation for the improvement of camel milk yield and quality, and providing important insights into the initiation and maintenance of the physiological process of lactation in camels.

### Supplementary Information

**Additional file 1: Supplementary Figure 1.** Sampling sites and camel photos.

**Additional file 2: Figure S2.** B-ultrasonic rectal examination of camels.

**Additional file 3: Supplementary Figure 3.** HE staining procedure.

**Additional file 4: Supplementary Figure 4.** PCA principal component analysis of samples.

**Additional file 5: Supplementary Figure 5.** Quantitative real-time PCR.

**Additional file 6: Supplementary Table 1.** Camel milk trait.

**Additional file 7: Supplementary Table 2.** Analysis of reads quality detection.

**Additional file 8: Supplementary Table 3.** Differentially expressed genes (WDL vs WGH).

**Additional file 9: Supplementary Table 4.** Differentially expressed genes (RSQ vs CRQ).

**Additional file 10: Supplementary Table 5.** Specific expression gene information.

**Additional file 11: Supplementary Table 6.** Animal QTL database milk traits related to genes.

**Additional file 12: Supplementary Table 7.** 1185 genes related to milk production traits in Alashan Bactrian Camel.

**Additional file 13: Supplementary Table 8.** Functional enrichment analysis (WDL vs WGH).

**Additional file 14: Supplementary Table 9.** WDLvsWGH 65 candidate genes.

**Additional file 15: Supplementary Table 10.** Functional enrichment analysis of 65 candidate genes (WDL vs WGH).

**Additional file 16: Supplementary Table 11.** Functional enrichment analysis (RSQ vs CRQ).

**Additional file 17: Supplementary Table 12.** RSQ vs CRQ 46 candidate genes.

**Additional file 18: Supplementary Table 13.** Functional enrichment analysis of 46 candidate genes (RSQ vs CRQ).

**Additional file 19: Supplementary Table 14.** Quantitative Real-time PCR data.

## Data Availability

The datasets used and/or analyzed during the current study are available from the corresponding author on reasonable request. Raw sequencing files have been deposited in the NCBI database (No. PRJNA892805 and PRJNA892714).

## Abbreviations

DEGs: Differentially expressed genes
RNA-seq: Ribonucleic acid sequencing
FPKM: Fragments per kilobase of transcript per million mapped reads
QTL: Quantitative trait locus
GO: Gene Ontology
KEGG: Kyoto Encyclopedia of Genes and Genomes
PPI: Protein–protein interaction
PCR: Polymerase chain reaction
qPCR: Quantitative real-time PCR
PCA: Principal component analysis

## References

[1] Dahiya SS, Nagarajan G, Bharti VK, Swami SK, Mehta SC, Tuteja FC, Narnaware SD, Patil N (2014). Sequence analysis of the Toll-like receptor 2 gene of old world camels. J Adv Res.

[2] Jirimutu, Wang Z, Ding G, Chen G, Sun Y, Sun Z, Zhang H, Wang L, Hasi S, Zhang Y (2012). Genome sequences of wild and domestic bactrian camels. Nat Commun.

[3] Ming L, Siren D, Hasi S, Jambl T, Ji R (2022). Review of genetic diversity in Bactrian camel (Camelus bactrianus). Anim Front.

[4] Shakeel K, Rabail R, Iahtisham Ul H, Sehar S, Nawaz A, Manzoor MF, Walayat N, Socol CT, Maerescu CM, Aadil RM (2022). Camel milk protectiveness toward multiple liver disorders: a review. Front Nutr.

[5] FAO. Dairy and dairy products OECD-FAO agricultural outlook 2020–2029; 2020.

[6] Sattar A. What is holding back milk production potential in Pakistan? PIDE Knowledge Brief. 2022:61–65.

[7] Bauman DE, Mather IH, Wall RJ, Lock AL (2006). Major advances associated with the biosynthesis of milk. J Dairy Sci.

[8] Farah Z (1993). Composition and characteristics of camel milk. J Dairy Res.

[9] Yang J, Dou Z, Peng X, Wang H, Shen T, Liu J, Li G, Gao Y (2019). Transcriptomics and proteomics analyses of anti-cancer mechanisms of TR35–an active fraction from Xinjiang Bactrian camel milk in esophageal carcinoma cell. Clin Nutr.

[10] Konuspayeva GS. Camel milk composition and nutritional value. In: Handbook of research on health and environmental benefits of camel products. Hershey: IGI global; 2020:15–40.

[11] Benmeziane-Derradji F (2021). Evaluation of camel milk: gross composition—a scientific overview. Trop Anim Health Pro.

[12] Farah Z, Mollet M, Younan M, Dahir R (2007). Camel dairy in Somalia: limiting factors and development potential. Livest Sci.

[13] Darwish AM, Abdelhafez MA, El-Metwaly HA, Khim JS, Allam AA, Ajarem JS. Genetic divergence of two casein genes and correlated milk traits in Maghrebi camels. Biologia. 2022;77(7):1889–98.

[14] Amandykova MDTA, Mussayeva AS, Saitou N (2021). Genotyping of camels of Almaty region by CSN2 dairy productivity gene. J Exp Biol.

[15] Jadhav SA, Umrikar UD, Sawane MP, Pawar VD, Mehta SC (2020). Genetic polymorphism at K-Casein gene in indian camel breeds (Camelus dromdarius). J Camel Pract Res.

[16] Yelubayeva ME, Buralkhiyev BA, Tyshchenko VI, Terletskiy VP, Ussenbekov YS (2018). Results of Camelus dromedarius and Camelus bactrianus Genotyping by Alpha-S1-Casein, Kappa-Casein Loci, and DNA Fingerprinting. Cytol Genet.

[17] Nowier AM, Ramadan SI (2020). Association of β-casein gene polymorphism with milk composition traits of Egyptian Maghrebi camels (Camelus dromedarius). Arch Anim Breed.

[18] Bai X, Zheng Z, Liu B, Ji X, Bai Y, Zhang W (2016). Whole blood transcriptional profiling comparison between different milk yield of Chinese Holstein cows using RNA-seq data. BMC Genomics.

[19] Bahbahani H, Musa HH, Wragg D, Shuiep ES, Almathen F, Hanotte O (2019). Genome diversity and signatures of selection for production and performance traits in dromedary camels. Front Genet.

[20] Sandri M, Stefanon B, Loor J (2015). Transcriptome profiles of whole blood in Italian Holstein and Italian Simmental lactating cows diverging for genetic merit for milk protein. J Dairy Sci.

[21] Cai J, Liang S, Xie Y, Zang X, Jiang L, Miao C, Liu J, Wang D. Milk yield variation partially attributed to blood oxygen-mediated neutrophil activation in lactating dairy goats. Brit J Nutr. 2023;129(3):369–80. 10.1017/S0007114522001015 35604023

[22] Sikka P, Singh KP, Singh I, Mishra DC, Paul SS, Balhara AK, Andonissamy J, Chaturvedi KK, Rao AR, Rai A (2023). Whole blood transcriptome analysis of lactating Murrah buffaloes divergent to contrasting genetic merits for milk yield. Front Vet Sci.

[23] Guo L, Lema D, Liu B, Dai L, Wang X, Wang X, Cao J, Zhang W. Identification of Bactrian camel milk-related genes and regulatory networks in supplementation and grazing. 2022. 10.1007/s11250-023-03749-3 37776405

[24] Wernery U (2006). Camel milk, the white gold of the desert. J Camel Pract Res.

[25] Demissie BE (2019). Assessment of artificial insemination in camel. J Anim Sci.

[26] Brinsko SP, Blanchard TL, Varner DD, Schumacher J, Love CC. Manual of equine reproduction. City of Saint Louis: Mosby; 2010.

[27] Skidmore JA (2015). The use of transrectal ultrasonography in camel reproduction Usingan Easi-Scan curve portable ultrasound machine. Arab World Agribusiness.

[28] Akers RM. Lactation and the mammary gland. New Jersey: Wiley-Blackwell; 2016.

[29] Richert MM, Schwertfeger KL, Ryder JW, Anderson SM (2000). An atlas of mouse mammary gland development. J Mammary Gland Biol Neoplasia.

[30] Mortazavi A, Williams BA, Mccue K, Schaeffer L, Wold B (2008). Mapping and quantifying mammalian transcriptomes by RNA-Seq. Nat Methods.

[31] Zhang J, Zhang Z, Liu W, Li L, Han L, Xu L, Zhao Y (2022). Transcriptome analysis revealed a positive role of Ethephon on chlorophyll metabolism of Zoysia japonica under Cold Stress. Plants (Basel).

[32] Gong X, Zheng M, Zhang J, Ye Y, Duan M, Chamba Y, Wang Z, Shang P (2022). Transcriptomics-based study of differentially expressed genes related to fat deposition in Tibetan and Yorkshire Pigs. Front Vet Sci.

[33] Ogata H, Goto S, Sato K, Fujibuchi W, Bono H, Kanehisa M (1999). KEGG: kyoto encyclopedia of genes and genomes. Nucleic Acids Res.

[34] Kanehisa M (2019). Toward understanding the origin and evolution of cellular organisms. Protein Sci.

[35] Kanehisa M, Furumichi M, Sato Y, Kawashima M, Ishiguro-Watanabe M (2023). KEGG for taxonomy-based analysis of pathways and genomes. Nucleic Acids Res.

[36] Zandi E, Mehrgardi AA, Esmailizadeh A (2020). Mammary tissue transcriptomic analysis for construction of integrated regulatory networks involved in lactogenesis of Ovis aries. Genomics.

[37] Szklarczyk D, Gable AL, Lyon D, Junge A, Wyder S, Huerta-Cepas J, Simonovic M, Doncheva NT, Morris JH, Bork P (2019). STRING v11: protein-protein association networks with increased coverage, supporting functional discovery in genome-wide experimental datasets. Nucleic Acids Res.

[38] Nangraj AS, Selvaraj G, Kaliamurthi S, Kaushik AC, Cho WC, Wei DQ (2020). Integrated PPI- and WGCNA-retrieval of hub gene signatures shared between Barrett’s Esophagus and esophageal adenocarcinoma. Front Pharmacol.

[39] Yu X, Wu Y, Zhang J, Zulipikaer A, Chen J (2020). Pre-evaluation of humoral immune response of Bactrian camels by the quantification of Th2 cytokines using real-time PCR. J Biomed Res.

[40] Schmittgen TD, Livak KJ (2008). Analyzing real-time PCR data by the comparative CT method. Nat Protoc.

[41] Purdy S (2002). Ultrasound examination of the female miniature donkey. N Engl J Large Anim Health.

[42] Ruan W, Monaco ME, Kleinberg DL (2005). Progesterone stimulates mammary gland ductal morphogenesis by synergizing with and enhancing insulin-like growth factor-I action. Endocrinology.

[43] Bao Z, Lin J, Ye L, Zhang Q, Chen J, Yang Q, Yu Q (2016). Modulation of mammary gland development and milk production by growth hormone expression in GH transgenic goats. Front Physiol.

[44] Liu L, Zhang Y, Ma H, Cao H, Liu W (2023). Integrating genome-wide methylation and transcriptome-wide analyses to reveal the genetic mechanism of milk traits in Kazakh horses. Gene.

[45] Liu L, Zhang Q (2019). Identification and functional analysis of candidate gene VPS28 for milk fat in bovine mammary epithelial cells. Biochem Biophys Res Commun.

[46] Hou Y, Xie Y, Yang S, Han B, Shi L, Bai X, Liang R, Dong T, Zhang S, Zhang Q (2021). EEF1D facilitates milk lipid synthesis by regulation of PI3K-Akt signaling in mammals. Faseb J.

[47] M H. Fat digestion and absorption. 2000.

[48] Yu X, Fang C, Liu L, Zhao X, Liu W, Cao H, Lv S (2021). Transcriptome study underling difference of milk yield during peak lactation of Kazakh horse. J Equine Vet Sci.

[49] Hao Z, Luo Y, Wang J, Hu J, Liu X, Li S, Jin X, Ke N, Zhao M, Hu L (2020). Rna-seq reveals the expression profiles of long non-coding rnas in lactating mammary gland from two sheep breeds with divergent milk phenotype. Animals.

[50] Wang Y, Guo W, Xu H, Tang K, Zan L, Yang W (2019). Melatonin suppresses milk fat synthesis by inhibiting the mTOR signaling pathway via the MT1 receptor in bovine mammary epithelial cells. J Pineal Res.

[51] He J, Zheng W, Xue Y, Guo H, Yao W (2018). 121 A controlled heat stress during late gestation affects thermoregulation, productive performance and metabolic profiles of primiparous sows. J Anim Sci.

[52] Li M, Li Q, Kang S, Cao X, Zheng Y, Wu J, Wu R, Shao J, Yang M, Yue X (2020). Characterization and comparison of lipids in bovine colostrum and mature milk based on UHPLC-QTOF-MS lipidomics. Food Res Int.

[53] Poisbeau P, Grinevich V, Charlet A, Hurlemann R, Grinevich V (2018). Oxytocin signaling in pain: cellular, circuit, system, and behavioral levels. Behavioral pharmacology of neuropeptides: oxytocin.

[54] Stelwagen K, Verkerk G, Phipps A, Matthews L (1997). Effect of cortisol on mammary tight junction (TJ) permeability in lactating dairy cows. Livest Prod Sci.

[55] Zeng H, Xia H, Wang X, Wang Y, Fang J, Li S, Zhai Y, Han Z (2023). Comprehensive profiling of ceRNA (circRNA-miRNA-mRNA) networks in hypothalamic-pituitary-mammary gland axis of dairy cows under heat stress. Int J Mol Sci.

[56] Miura H, Yamazaki T, Kikuchi M, Sakaguchi M (2019). Plasma steroid hormone concentrations and their relationships in Suffolk ewes during gestation and parturition. Anim Sci J.

[57] Kozlowski CP, Bauman KL, Clawitter HL, Hall R, Poelker C, Thier T, Fischer M, Powell DM (2022). Noninvasive monitoring of steroid hormone production and activity of zoo-housed banteng (Bos javanicus). Anim Reprod Sci.

[58] Salcedo-Tacuma D, Parales-Giron J, Prom C, Chirivi M, Laguna J, Lock AL, Contreras GA (2020). Transcriptomic profiling of adipose tissue inflammation, remodeling, and lipid metabolism in periparturient dairy cows (Bos taurus). BMC Genomics.

[59] Nagel C, Aurich C, Aurich J (2019). Stress effects on the regulation of parturition in different domestic animal species. Anim Reprod Sci.

[60] Zhang Z, Wei Q, Zeng Y, Jia X, Su H, Lin W, Xing N, Bai H, He Y, Wang Q (2021). Effect of Hordei Fructus Germinatus on differential gene expression in the prolactin signaling pathway in the mammary gland of lactating rats. J Ethnopharmacol.

[61] Gherissi DE, Afri-Bouzebda F, Bouzebda Z, Bonnet X (2018). Are female camels capital breeders? Influence of seasons, age, and body condition on reproduction in an extremely arid region. Mamm Biol.

[62] Mastrorocco A, Ludovica C, Martino NA, Camillo F, Diana F, Elena C, Lacalandra GM, Roelen BA, Arti A, Dell’Aquila ME. Alginate engineered cumulus oocyte enclosing microbeads for modelling 3D oocyte maturation. In: European College of Animal Reproduction; 2019:97–97.

[63] Do DN, Li R, Dudemaine PL, Ibeagha-Awemu EM (2017). MicroRNA roles in signalling during lactation: an insight from differential expression, time course and pathway analyses of deep sequence data. Sci Rep.

[64] Theil PK, Farmer C, Feyera T (2022). Physiology and nutrition of late gestating and transition sows. J Anim Sci.

[65] Singh KV, Jayakumar S, Dixit S, Malik Z (2019). Molecular characterization and genetic variability of Alpha Casein gene, CSN1S1 in Bikaneri camel (Camelus dromedarius) milk. Indian J Anim Res.

[66] Hemati B, Banabazi M, Shahkarami S, Mohandesan E, Burger P (2017). Genetic diversity within Bactrian camel population of Ardebil province. Res Anim Prod.

[67] Al-Sharif MM, Radwan HA, Hendam BM, Ateya AI (2022). Exploring single nucleotide polymorphisms in GH, IGF-I, MC4R and DGAT1 genes as predictors for growth performance in dromedary camel using multiple linear regression analysis. Small Ruminant Res.

[68] Maulana MBTA, Widayanti S, Vanessa R, Pambuko G, Lestari REP, Herowati N, Gunawan T, Prastowo S, Susilawati A (2022). Sutarno: association of Lactoferrin (LTF) gene variation to milk yield in Indonesian Friesian Holstein. IOP Conf Ser: Earth Environ Sci.

[69] Yakan A, Ozkan H, Eraslan A, Ünal N, Özbeyaz C (2018). Gene expression levels in some candidate genes for mastitis resistance, milk yield, and milk quality of goats reared under different feeding systems. Turk J Vet Anim Sci.

[70] Li Y, Han B, Liu L, Zhao F, Liang W, Jiang J, Yang Y, Ma Z, Sun D (2019). Genetic association of DDIT3, RPL23A, SESN2 and NR4A1 genes with milk yield and composition in dairy cattle. Anim Genet.

[71] Johnston D, Malo Estepa I, Ebhardt H, Crowe M, Diskin M. Identification of milk whey protein biomarkers of early pregnancy status in dairy cattle. 2018.

[72] Getaneh M, Alemayehu K (2022). Candidate genes associated with economically important traits in dairy goats. Cogent Food Agric.

[73] Naserkheil M, Mehrban H, Lee D, Park MN (2021). Genome-wide association study for carcass primal cut yields using single-step Bayesian approach in Hanwoo cattle. Front Genet.

[74] Lopes-Marques M, Ruivo R, Alves LQ, Sousa N, Machado AM, Castro LFC (2019). The singularity of cetacea behavior parallels the complete inactivation of melatonin gene modules. Genes (Basel).

[75] Raven L-A, Cocks BG, Goddard ME, Pryce JE, Hayes BJ (2014). Genetic variants in mammary development, prolactin signalling and involution pathways explain considerable variation in bovine milk production and milk composition. Genet Sel Evol.

[76] Liu L, Sun B, Zhang F, Zhong Z, Zhang Y, Li F, Zhang T, Khatib H, Wang X (2022). lncRNA MPFAST promotes proliferation and fatty acid synthesis of bovine mammary epithelial cell by sponging miR-103 regulating PI3K-AKT pathway. J Agr Food Chem.

[77] Bhat SA, Ahmad SM, Ibeagha-Awemu EM, Bhat BA, Dar MA, Mumtaz PT, Shah RA, Ganai NA (2019). Comparative transcriptome analysis of mammary epithelial cells at different stages of lactation reveals wide differences in gene expression and pathways regulating milk synthesis between Jersey and Kashmiri cattle. PLoS One.

[78] Fata JE, Mori H, Ewald AJ, Zhang H, Yao E, Werb Z, Bissell MJ (2007). The MAPKERK-1, 2 pathway integrates distinct and antagonistic signals from TGFα and FGF7 in morphogenesis of mouse mammary epithelium. Dev Biol.

[79] Wu M, Qiu Q, Zhou Q, Li J, Yang J, Zheng C, Luo A, Li X, Zhang H, Cheng X (2022). circFBXO7/miR-96-5p/MTSS1 axis is an important regulator in the Wnt signaling pathway in ovarian cancer. Mol Cancer.

[80] Asadi Yousefabad SL, Tamadon A, Rahmanifar F, Jafarzadeh Shirazi MR, Sabet Sarvestani F, Tanideh N, Abli Moghadam AA, Niazi A (2013). Lactation effect on the mRNAs expression of RFRP-3 and KiSS-1 in dorsomedial and arcuate nuclei of the rat hypothalamus. J Physiol Pharmacol.

[81] da Cruz AS, Silva DC, Minasi LB, de Farias Teixeira LK, Rodrigues FM, da Silva CC, do Carmo AS, da Silva MVGB, Utsunomiya YT, Garcia JF et al. Single-nucleotide polymorphism variations associated with specific genes putatively identified enhanced genetic predisposition for 305-day milk yield in the Girolando crossbreed. Front Genet. 2021;11:344. 10.3389/fgene.2020.573344 PMC7876550 33584786

[82] Iovino M, Messana T, Tortora A, Giusti C, Lisco G, Giagulli VA, Guastamacchia E, De Pergola G, Triggiani V (2021). Oxytocin signaling pathway: from cell biology to clinical implications. Endocr Metab Immune Disord Drug Targets.

[83] Lacouture A, Jobin C, Weidmann C, Berthiaume L, Bastien D, Laverdière I, Pelletier M, Audet-Walsh É (2021). A FACS-free purification method to study estrogen signaling, organoid formation, and metabolic reprogramming in mammary epithelial cells. Front Endocrinol.

[84] Foyouzi N, Frisbæk Y, Norwitz ER (2004). Pituitary gland and pregnancy. Obstetr Gynecol Clinics.

[85] Alex A, Bhandary E, McGuire KP, Alipour S, Omranipour R (2020). Anatomy and physiology of the breast during pregnancy and lactation. Diseases of the breast during pregnancy and lactation.

