# Supplementary material for: Transcriptome analysis of the Bactrian camel (Camelus bactrianus) reveals candidate genes affecting milk production traits
Source: BMC Genomics. 2023 Nov 2;24:660. doi: 10.1186/s12864-023-09703-9 (PMC10621195; doi:10.1186/s12864-023-09703-9)

Supplementary figure 1 Sampling sites and camel photos

Sampling sites

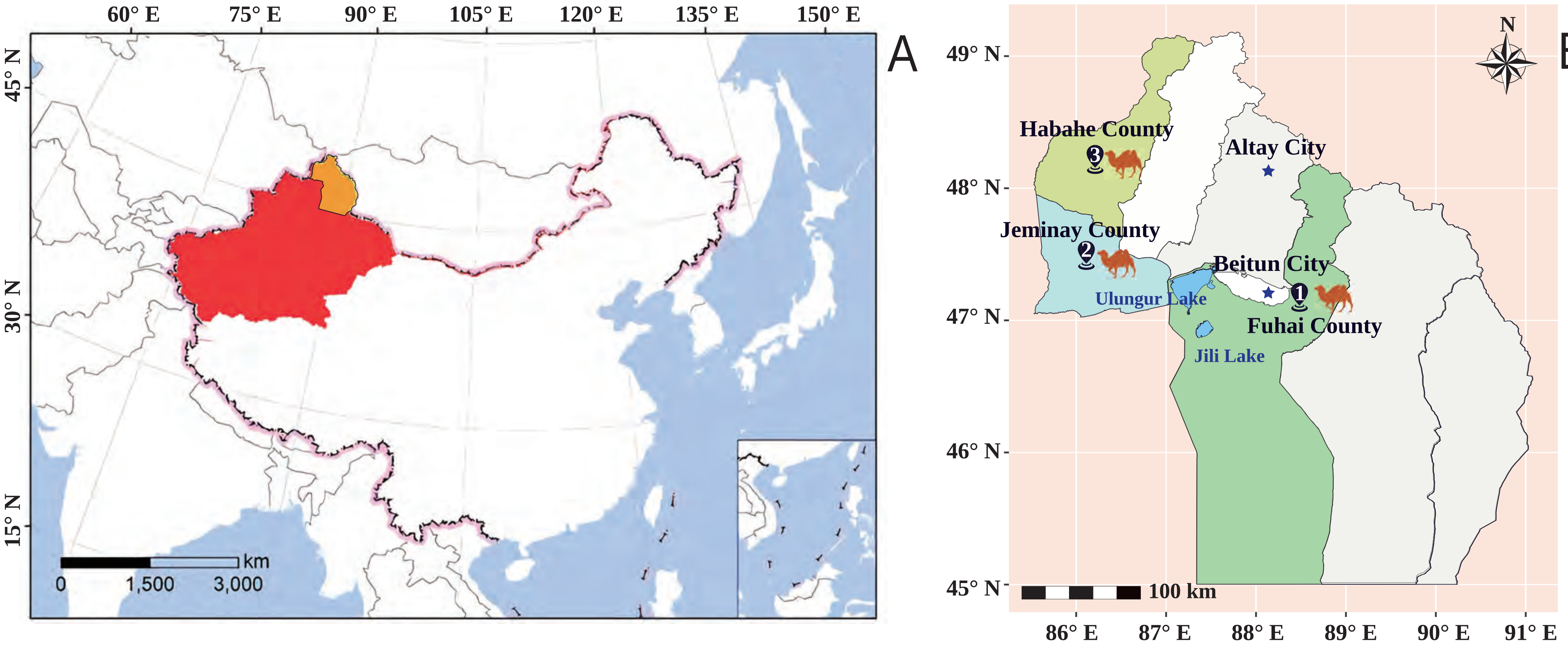

Camel photoes

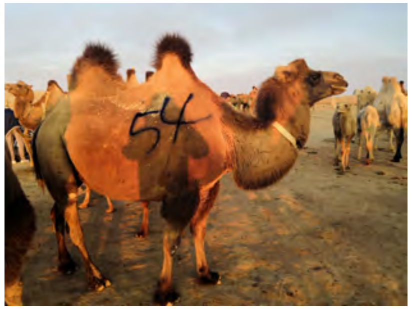

WGH1

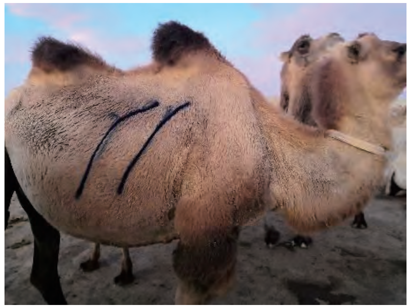

WGH2

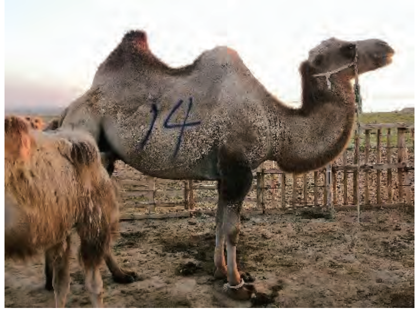

WGH3

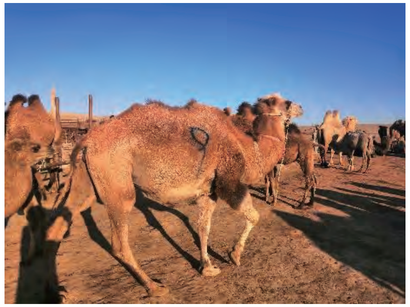

WDL1

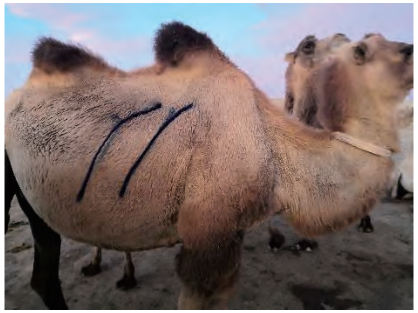

WDL2

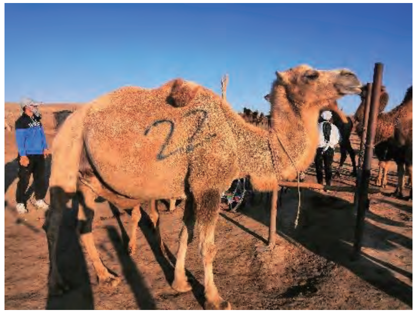

WDL3

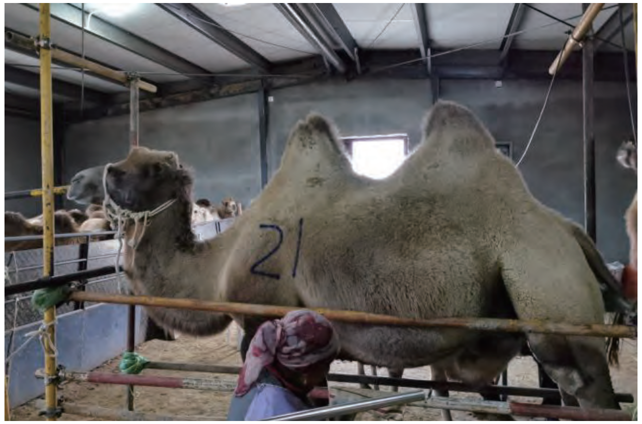

RSQ/CRQ1

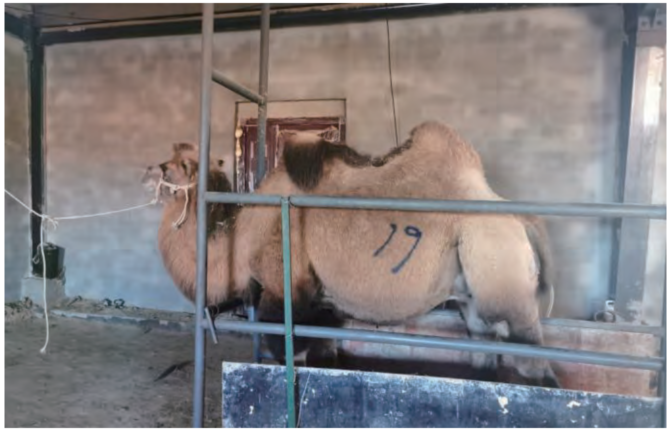

RSQ/CRQ2

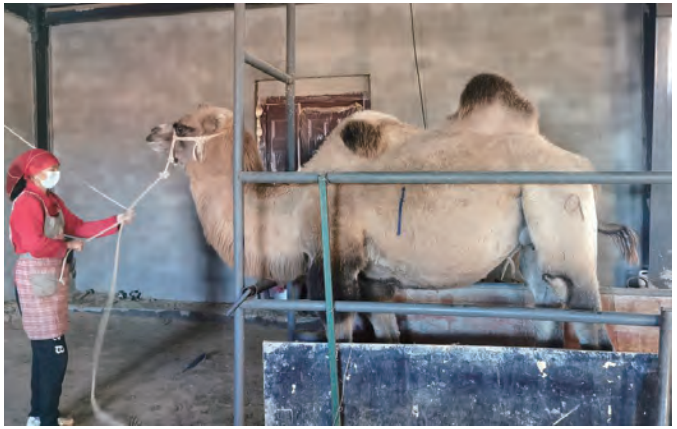

RSQ/CRQ3

Partial sample photos	
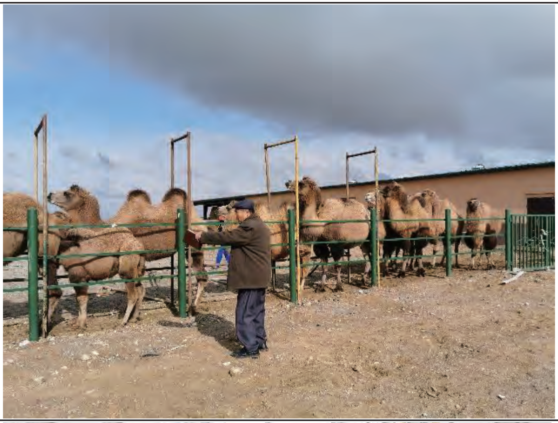	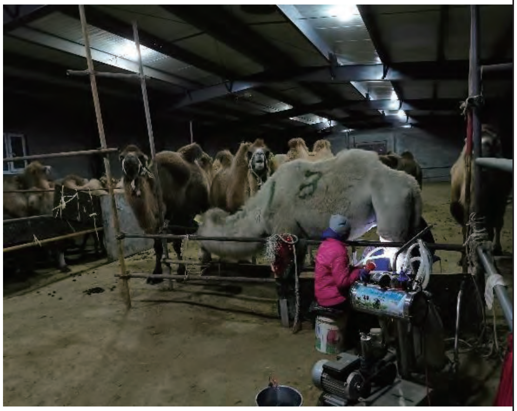
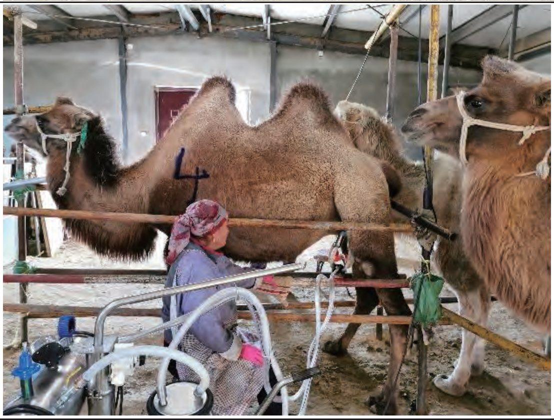	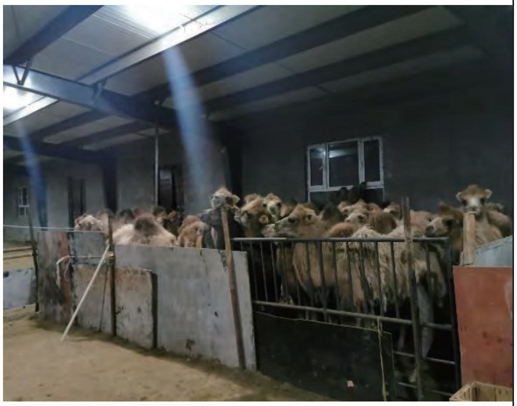
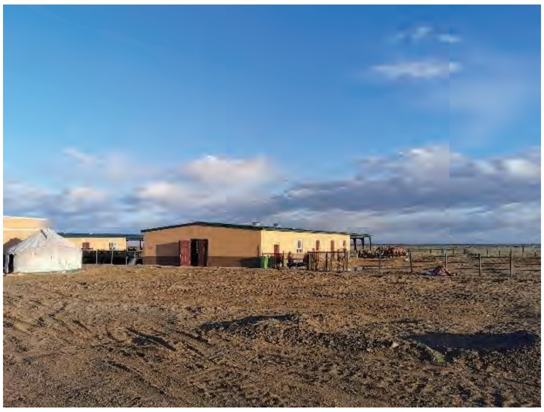	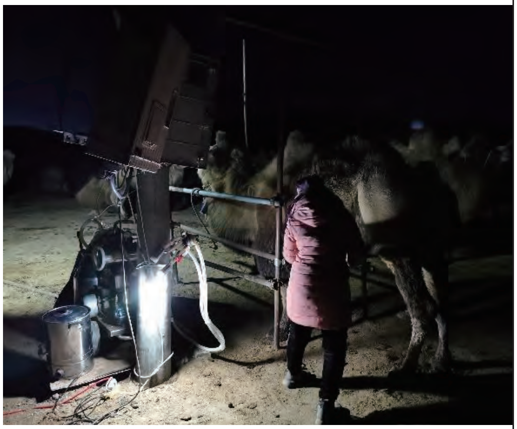
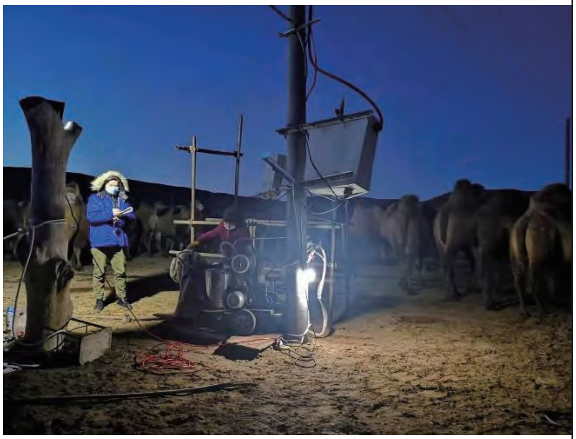	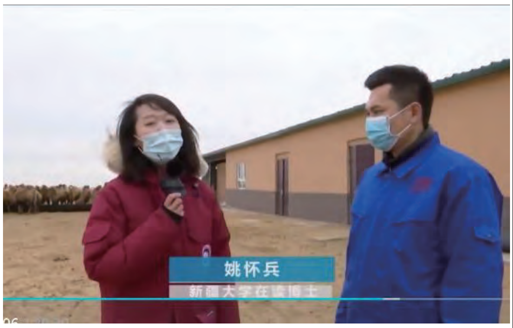

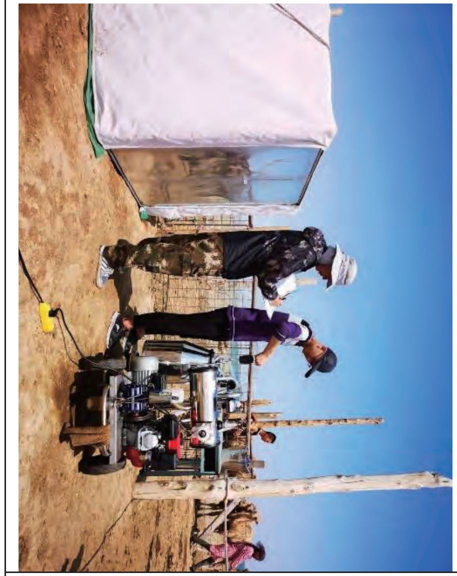
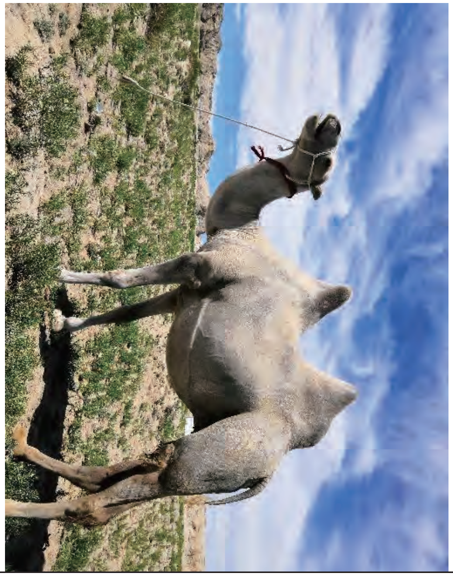
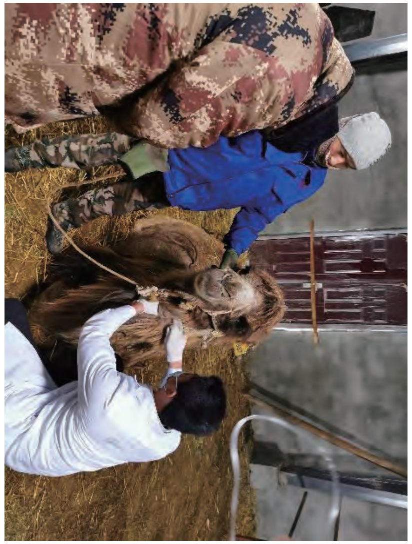
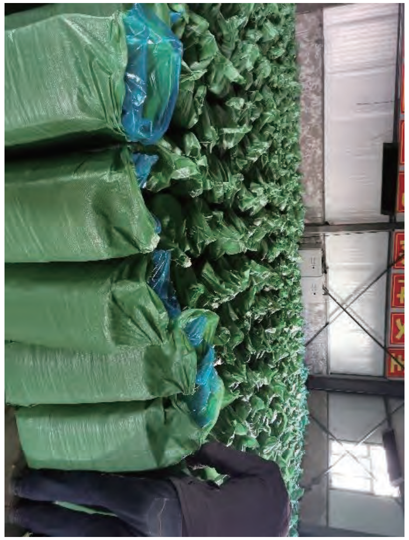
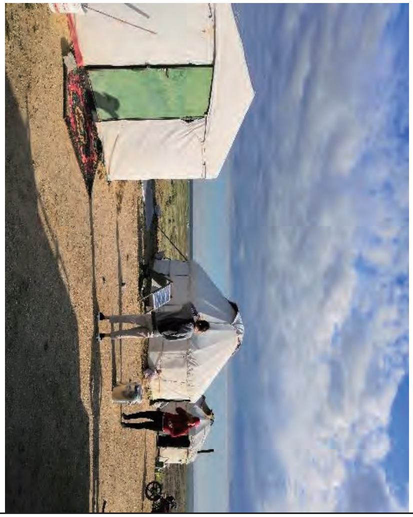
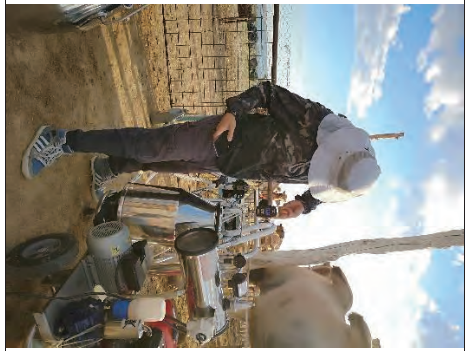
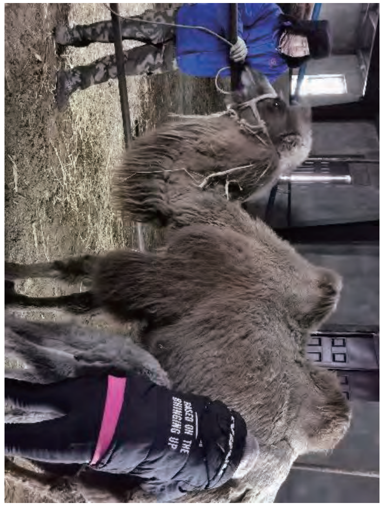
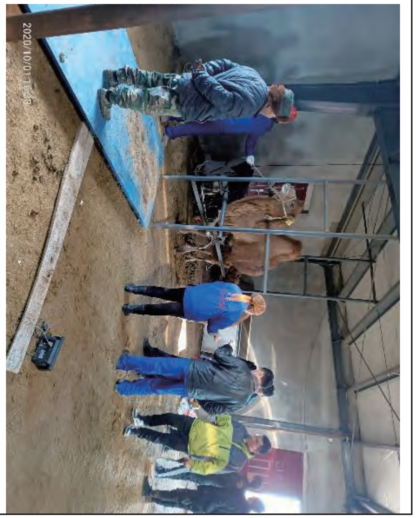

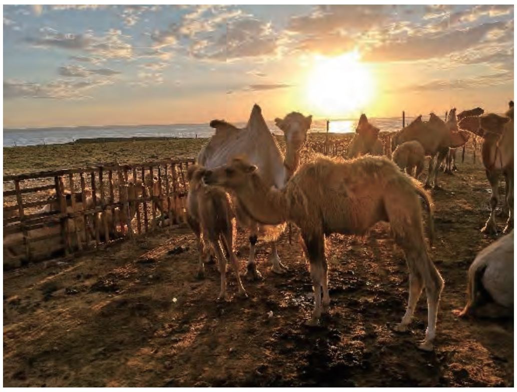
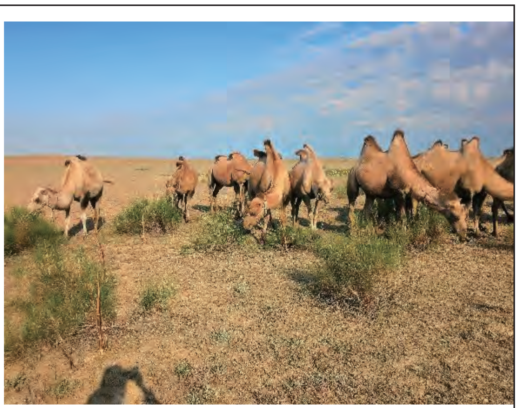
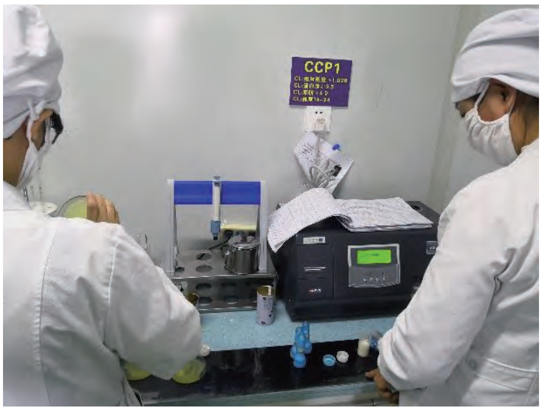
CCP1
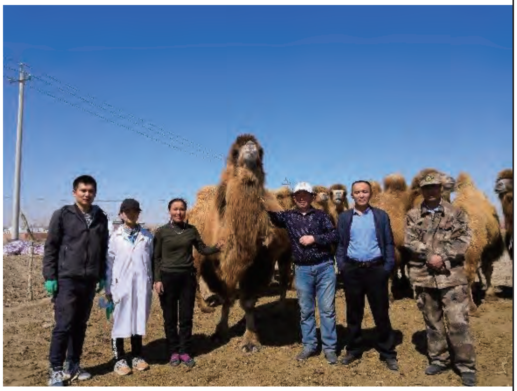
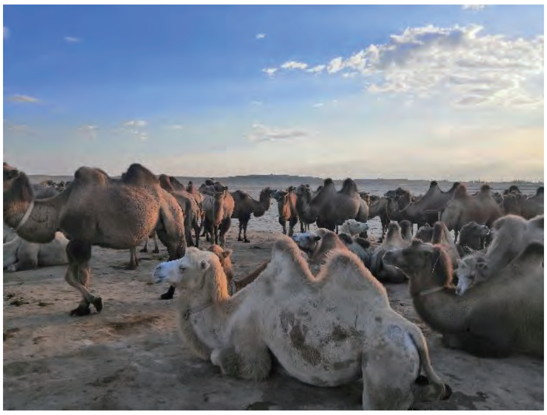
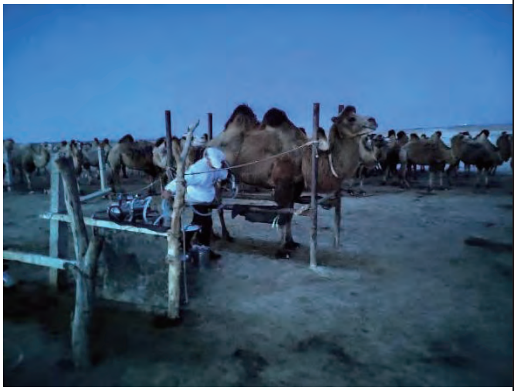
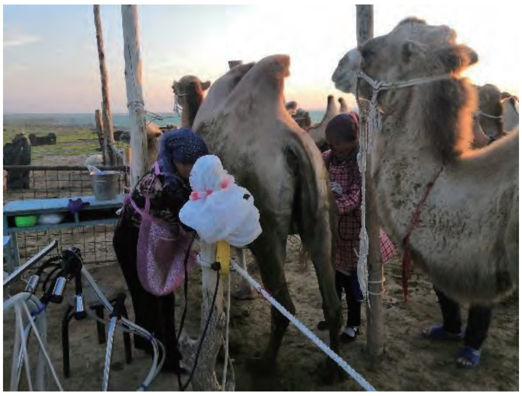
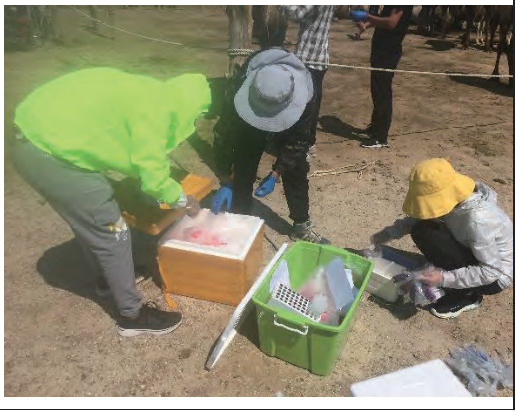

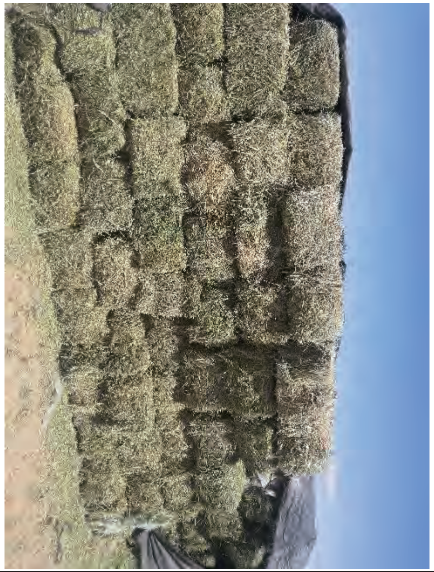
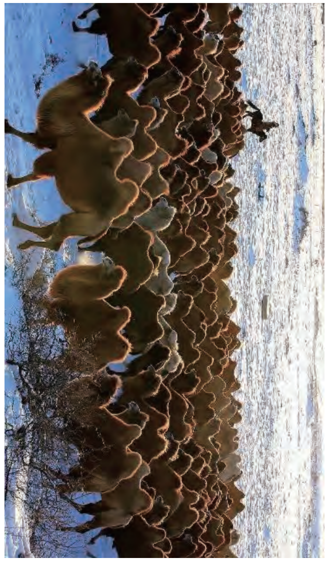
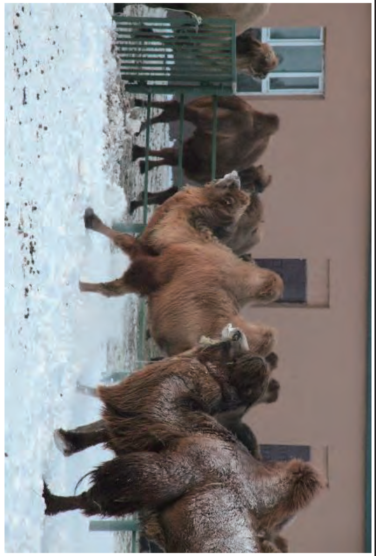
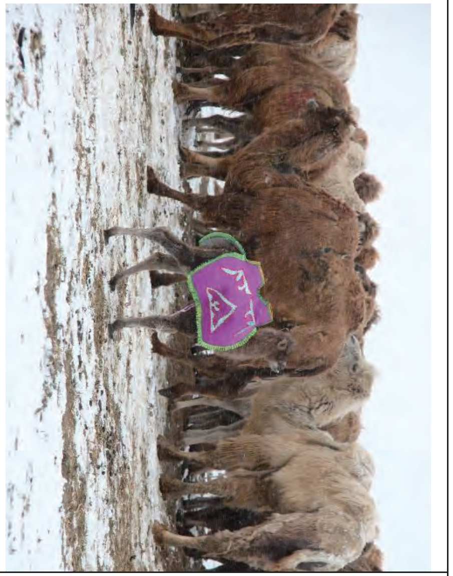
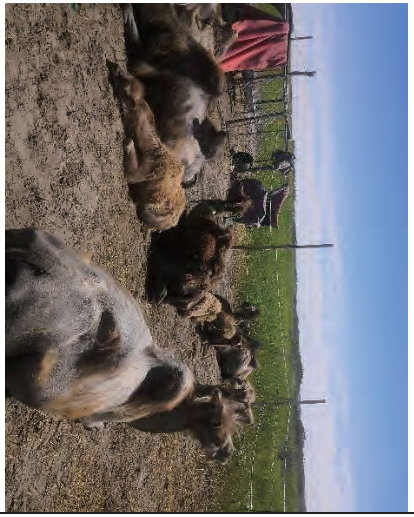
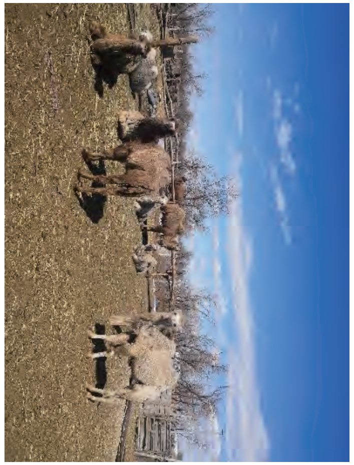
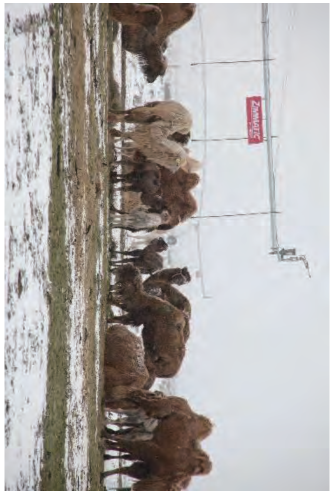
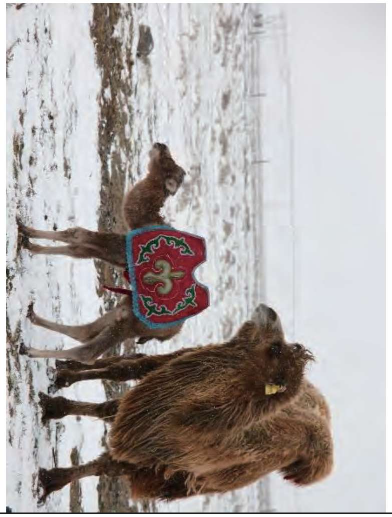

Supplement: Supplementary file 1 — Additional file 1: Supplementary Figure 1. Sampling sites and camel photos. [file 12864_2023_9703_MOESM1_ESM.pdf]
