# Supplementary material for: Transcriptome analysis of the Bactrian camel (Camelus bactrianus) reveals candidate genes affecting milk production traits
Source: BMC Genomics. 2023 Nov 2;24:660. doi: 10.1186/s12864-023-09703-9 (PMC10621195; doi:10.1186/s12864-023-09703-9)
Supplement: Supplementary file 2 — Additional file 2: Figure S2. B-ultrasonic rectal examination of camels. [file 12864_2023_9703_MOESM2_ESM.pdf]

Figure S2 B-ultrasonic rectal examination of camels

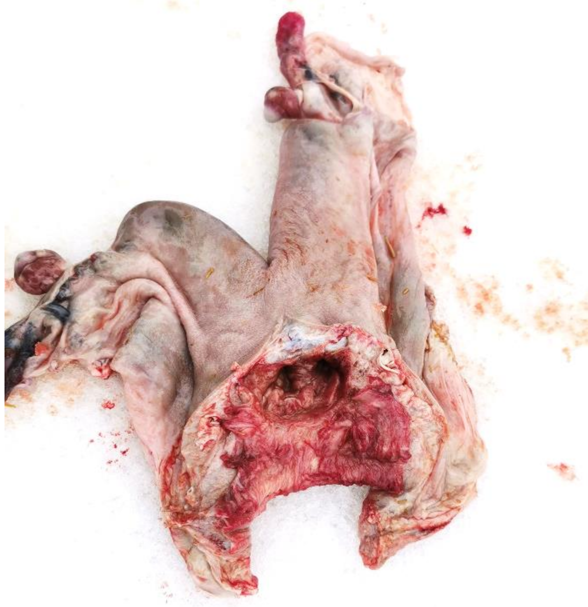

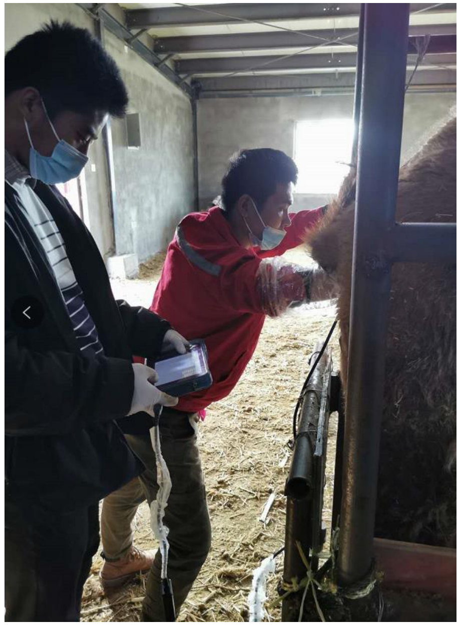

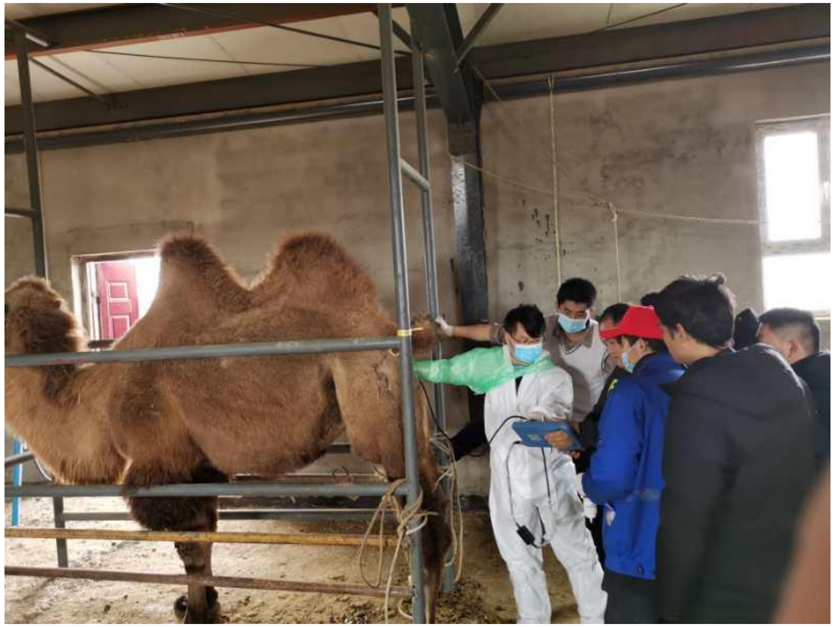

Dorsal view of the male reproductive tract

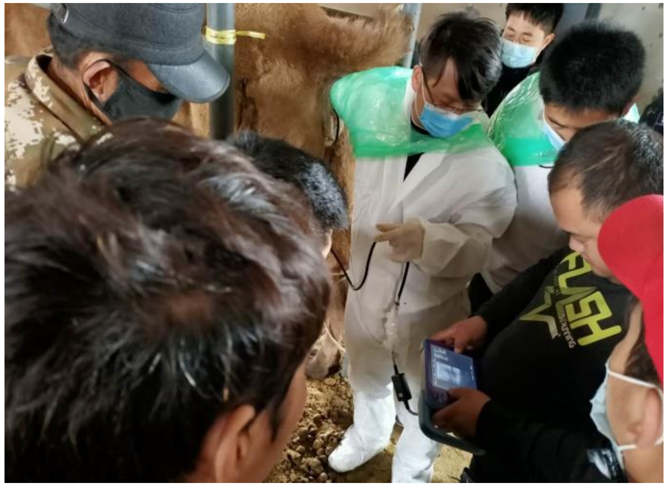

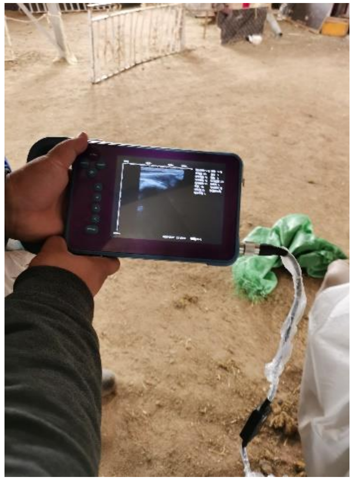

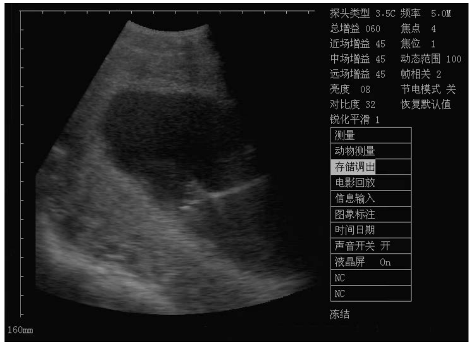

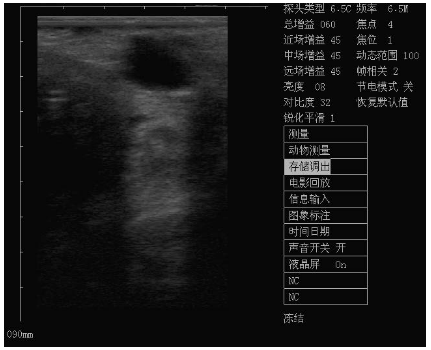

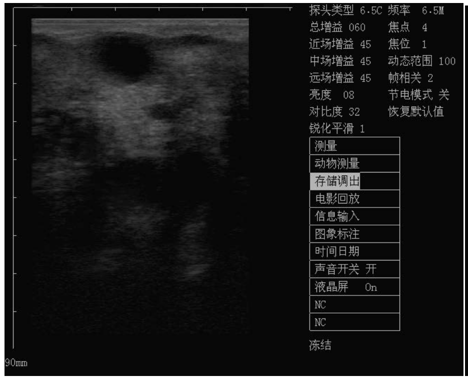

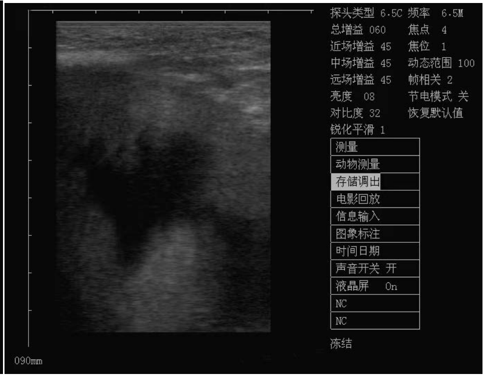

Examiner moved the ultrasound transducer to the tip of the uterine horn and ovary

https://baijiahao.baidu.com/s?id=1682562489186103966&wfr=spider&for=pc

https://new.qq.com/rain/a/20201106A0GORZ00
