# Supplementary material for: Transcriptome analysis of the Bactrian camel (Camelus bactrianus) reveals candidate genes affecting milk production traits
Source: BMC Genomics. 2023 Nov 2;24:660. doi: 10.1186/s12864-023-09703-9 (PMC10621195; doi:10.1186/s12864-023-09703-9)
Supplement: Supplementary file 3 — Additional file 3: Supplementary Figure 3. HE staining procedure. [file 12864_2023_9703_MOESM3_ESM.pdf]

Supplementary figure 3 HE staining procedure

1 Apparatus and reagents

1.1 Major apparatus

Name	Producer	Model
Dehydrator	DIAPATH	Donatello
Embedding machine	Wuhan Junjie Electronics Co., Ltd	JB-P5
Pathology slicer	Leica	RM2016
Frozen platform	Wuhan Junjie Electronics Co., Ltd	JB-L5
Organizer	KEDEE	KD-P
Dyeing machine	DIAPATH	Giotto
oven	Labotery	GFL-230
Glass slide	Servicebio	G6004
Upright optical microscope	Nikon	NIKON ECLIPSE E100
Imaging system	Nikon	NIKON DS-U3

1.2 Major reagents

Name	Producer	Code
Ethanol	SCRC	100092683
Xylene	SCRC	10023418
HE dye solution set	Servicebio	G1003
Neutral gum	SCRC	10004160

2 Procedure

2.1 Dewaxing as followed:

Xylene I for 20 min;

Xylene II for 20 min;

100% ethanol I for 5 min;

100% ethanol II for 5 min;

75% ethanol for 5 min;

Rinsing with tap water ;

2.2 Stain sections with Hematoxylin solution for 3-5 min, rinse with tap water. Then treat the section with Hematoxylin Differentiation solution, rinse with tap water. Treat the section with Hematoxylin Scott Tap Bluing, rinse with tap water.

2.3 85% ethanol for 5 min; 95% ethanol for 5 min; Finally Stain sections with Eosin dye for 5 min.

2.4 Dehydrate as followed:

100% ethanol I for 5 min;

100% ethanol II for 5 min;

100% ethanol III for 5 min;

Xylene I for 5 min;

Xylene II for 5 min;

Finally seal with neutral gum.

2.5 Observe with microscope inspection, image acquisition and analysis.

3 Results

Color	Result
Blue	Nucleus
Red	Cytoplasm

4 Precautions

4.1 Pay attention to the degree of cell differentiation;

Pay attention to the potency of hematoxylin and eosin, and change the dyeing solution in time.
