# Supplementary figures and images for: Transcriptome analysis of the Bactrian camel (Camelus bactrianus) reveals candidate genes affecting milk production traits
Source: BMC Genomics. 2023 Nov 2;24:660. doi: 10.1186/s12864-023-09703-9 (PMC10621195; doi:10.1186/s12864-023-09703-9)

Supplementary figure 4 PCA principal component analysis of samples

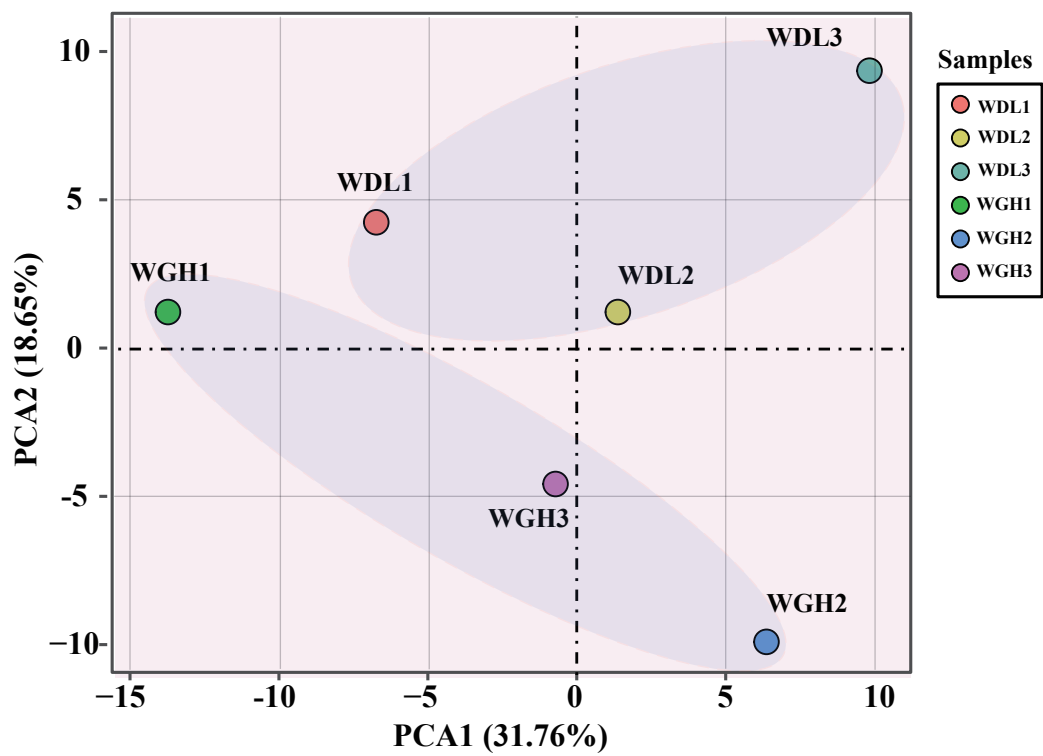

WDL VS WGH

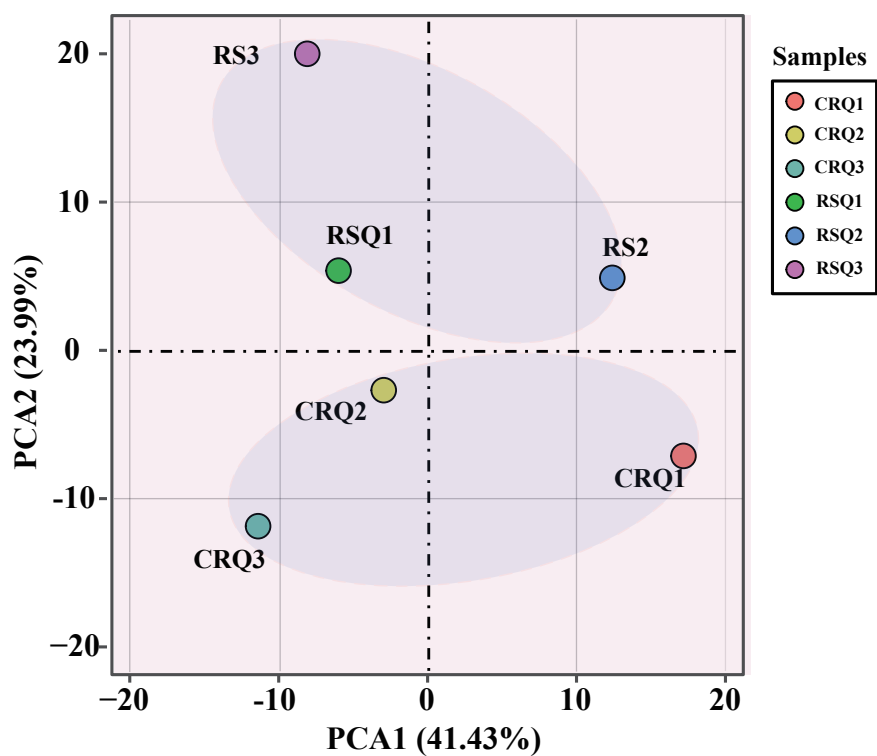

RSQ VS CRQ

Supplement: Supplementary file 4 — Additional file 4: Supplementary Figure 4. PCA principal component analysis of samples. [file 12864_2023_9703_MOESM4_ESM.pdf]

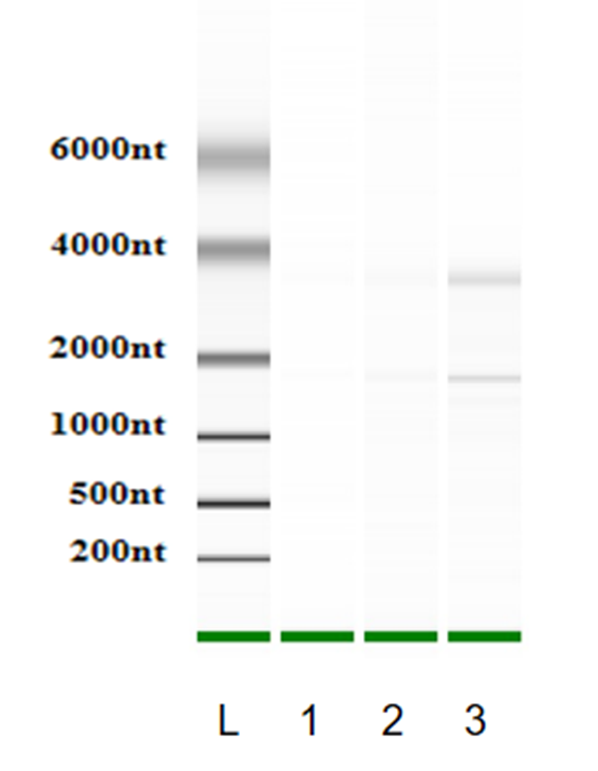

Supplement: Supplementary file 5 — Additional file 5: Supplementary Figure 5. Quantitative real-time PCR. [file 12864_2023_9703_MOESM5_ESM.zip › Supplementary figure 5 Total RNA agarose gel electrophoresis.png]
