# Supplementary material for: Transcriptome analysis of the Bactrian camel (Camelus bactrianus) reveals candidate genes affecting milk production traits
Source: BMC Genomics. 2023 Nov 2;24:660. doi: 10.1186/s12864-023-09703-9 (PMC10621195; doi:10.1186/s12864-023-09703-9)

Supplementary figure 5 Quantitative Real-time PCR

Total RNA agarose gel electrophoresis
（Take one of the samples as an example）

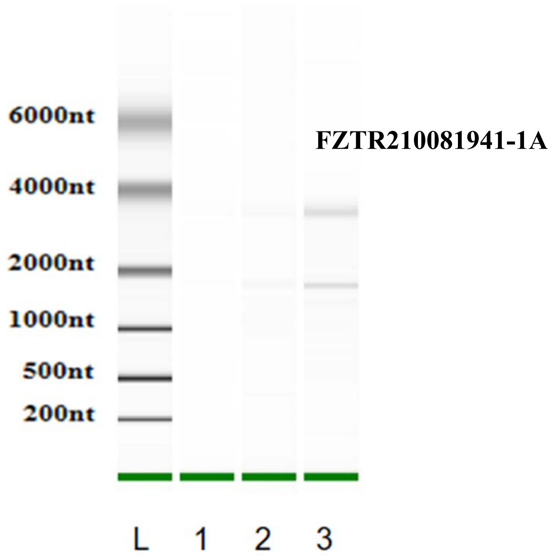

RNA integrity testing
（Take one of the samples as an example）

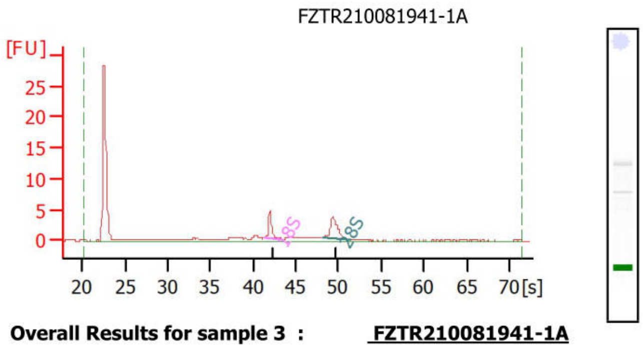

Overall Results for sample 3 : FZTR210081941-1A

RNA Area:	35.2
RNA Concentration:	18 ng/µl
rRNA Ratio [28s / 18s]:	1.5
RNA Integrity Number (RIN):	8.4 (B.02.10)
Result Flagging Color:	
Result Flagging Label:	RIN: 8.40

Fragment table for sample 3 : FZTR210081941-1A

Name	Start Time [s]	End Time [s]	Area	% of total Area
18S	41.37	43.60	4.7	13.3
28S	48.21	51.27	7.0	20.0

Reverse transcription steps

Genomic DNA removal: add each component according to the table below and incubate at 37°C for 30 minutes and then inactivate DNase I at 65°C for 10 minutes.

Components	*Volume*
RNA	*1ug*
10×DNase Buffer	*1uL*
DNase I	*1 uL*
Ribolock ™ Ribonuclease Inhibitor（40u/ul）	*0.25 uL*
RNase free water	*Up to 10 uL*

Reverse transcription.

1. Add the following reagents to the PCR tube.

Components	***Volume***
RNA	X µl（约 1ug）
Random6 Primer(50um)	2µl
RNase free H2O	Add to 12.5µl
Total	**12.5µl**

2. Mix gently, denature at 65℃ for 5min, then immediately put on ice for 2min.

Components	***Volume***
RNA-primer mix	**12.5µl**
5×RT Reaction Buffer	**4µl**
dNTP（10mM）	**2µl**
RNasin RNA	**0.5µl**
RevertAid Reverse Transcriptase	**1µl**
Total	**20µl**

Incubate at 25°C for 10 min, then incubate at 42°C for 60 min and heat inactivate at 70°C for 10 min. store at -20°C.

Real-time PCR

Components	***Volume***
AceQ Universal SYBR qPCR Master Mix	10µl
**Forward**	0.4uL
**Reverse**	0.4uL
**ddH$_2$O**	6.7µl
**cDNA**	2.5µl
**Total**	20µl

Reaction conditions

**95°C**	**5min**	
**95°C**	**10s**	**40 cycles**
**60°C**	**30s**	

Amplification procedure diagram: (with an annealing temperature of

60°C as an example)

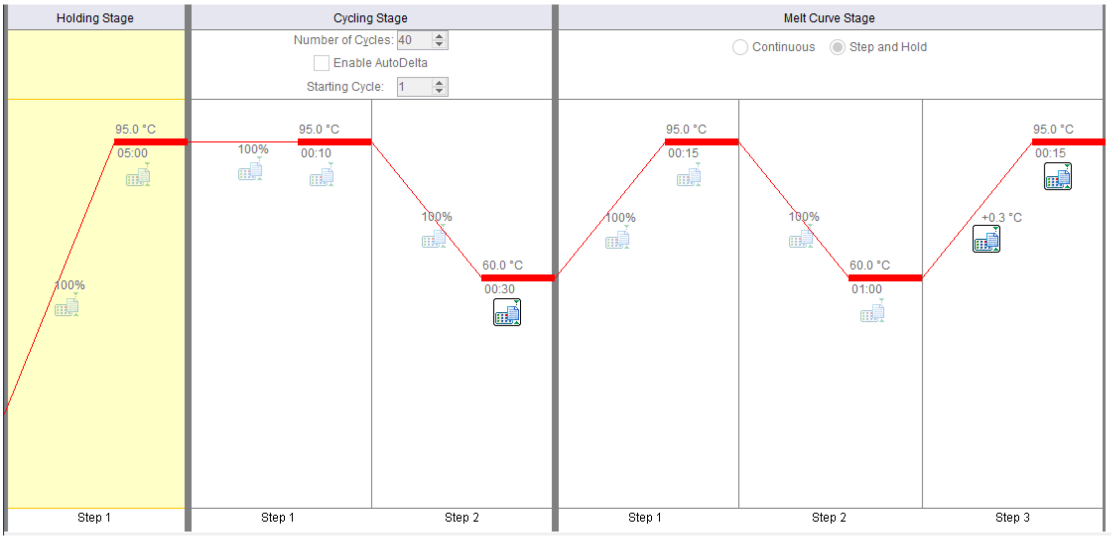

Supplement: Supplementary file 5 — Additional file 5: Supplementary Figure 5. Quantitative real-time PCR. [file 12864_2023_9703_MOESM5_ESM.zip › Supplementary figure 5 Quantitative Real-time PCR.pdf]
